# CD13 expression affects glioma patient survival and influences key functions of human glioblastoma cell lines in vitro

**DOI:** 10.1186/s12885-024-12113-z

**Published:** 2024-03-22

**Authors:** Wenying Zhang, Anne Blank, Irina Kremenetskaia, Anja Nitzsche, Güliz Acker, Peter Vajkoczy, Susan Brandenburg

**Affiliations:** 1 Department of Experimental Neurosurgery, Charité– Universitätsmedizin Berlin, corporate member of Freie Universität Berlin and Humboldt-Universität zu Berlin, Charitéplatz 1, 10117 Berlin, Germany; 2 Department of Neurosurgery, Charité– Universitätsmedizin Berlin, corporate member of Freie Universität Berlin and Humboldt-Universität zu Berlin, Charitéplatz 1, 10117 Berlin, Germany; 3 Berlin Institute of Health at Charité, Universitätsmedizin Berlin, Charitéplatz 1, 10117 Berlin, Germany

**Keywords:** Glioblastoma, Bioinformatics, Aminopeptidases, U118, U1242, T98G, SF188

## Abstract

CD13 (APN) is an Alanyl-Aminopeptidase with diverse functions. The role of CD13 for gliomas is still unknown. In this study, data of glioma patients obtained by TCGA and CGGA databases were used to evaluate the survival rate and prognostic value of CD13 expression level. Protein expression of CD13 was confirmed by immunofluorescence staining of fresh patient tissues. Eight human glioblastoma cell lines were studied by RT-PCR, Western Blot, immunofluorescence staining and flow cytometry to define CD13 expression. Cell lines with different CD13 expression status were treated with a CD13 inhibitor, bestatin, and examined by MTT, scratch and colony formation assaysas well as by apoptosis assay and Western Blots. Bioinformatics analysis indicated that patients with high expression of CD13 had poor survival and prognosis. Additionally, CD13 protein expression was positively associated with clinical malignant characteristics. Investigated glioblastoma cell lines showed distinct expression levels and subcellular localization of CD13 with intracellular enrichment. Bestatin treatment reduced proliferation, migration and colony formation of glioma cells in a CD13-dependent manner while apoptosis was increased. In summary, CD13 has an impact on glioma patient survival and is important for the main function of specific glioma cells.

**Supplementary Information:**

The online version contains supplementary material available at 10.1186/s12885-024-12113-z.

## Introduction

Gliomas account for approximately 80% of malignant brain tumors in adults, with glioblastoma (GBM) being the most common malignant glioma classified as WHO grade IV. For GBM, the incidence increases after the age of 40 and peaks at around 80 [1, 2]. Patients commonly experience headache and fast growing tumors increase the intracranial pressure leading to seizures, nausea and vomiting as well as fatigue [3]. The standard of care includes total resection of tumor tissue followed by radiation and chemotherapy [4–6]. However, GBM remains incurable, with an overall survival of only 15 months [5, 7]. In the last decades, various therapeutic strategies were investigated to overcome resistance development of GBM but with limited success [2]. Thus, new therapeutic approaches are required.

CD13 (Aminopeptidase N, APN) is an ectoenzyme that functions as a type II integral membrane protein (∼ 160 kDa). Additionally, cytoplasmic isoforms (110-130 kDa) have been identified, which may serve as intracellular precursors of the membrane-bound isoform [8, 9]. CD13 exhibits several functions as an enzyme, receptor and signaling molecule involved in peptide cleavage, endocytosis, differentiation, proliferation, angiogenesis, adhesion, chemotaxis and phagocytosis [10]. This protein is expressed by monocytes/macrophages [11], pericytes [12] and activated endothelial cells [13]. Furthermore, CD13 has been detected in many tumor entities, influencing their progression, prognosis, and sensitivity to chemotherapy [10, 14–16]. While leukemia, ovarian, renal and prostate cancer cells showed increased proliferation with CD13 overexpression [17–20], gastric cancer cells exhibited reduced proliferative activity in the presence of CD13 [21]. Thus, the function of CD13 appears to be context-dependent [15].

In the brain, CD13 is highly expressed in specific brain regions, such as choroid plexus, cortex, thalamus and spinal cord, where it is involved in metabolism of neuropeptides (e.g. enkephalins) by modifying signal transduction [22, 23]. Our understanding of CD13 in relation to GBM is limited, with only some indications of its expression in glioblastoma tissues [24, 25]. It was shown that tumor-associated microglia/macrophages (TAMs) express CD13 in a murine glioma model [26] and should be relevant for their phagocytic activity [27]. Additionally, TAMs and granulocytes express CD13 in human GBM specimen [28], implicating immune cells might constitute a considerable portion of the overall expression of CD13.

There are attempts to inhibit CD13 using aminopeptidase inhibitors like bestatin. Bestatin was used for diverse in vitro and in vivo cancer studies. It was shown that bestatin inhibits the proliferation and migration of several tumor cells and affects tumor growth [17–20, 29, 30]. Consequently, this inhibitor has been investigated as a therapeutic approach, both alone and in combination with other drugs [29–31]. Furthermore, bestatin acts as a radiosensitizer, enhancing the efficacy of radiotherapy in the context of cervical cancer, as demonstrated in a nude mouse tumor model [32]. Clinical trials reveal the potential of bestatin as an immunomodulator in leukemia and solid tumors after chemotherapy or radiotherapy [33–36]. This promising capability suggests a role for bestatin in augmenting the body’s immune response against cancer cells. Overall, bestatin is a well-described CD13 inhibitor, but has never been used to modulate CD13 function in glioblastoma cells.

Due to missing data about CD13 expression in GBM specimens and cells, we analyzed databases, tissues of GBM patients, and eight glioblastoma cell lines concerning the expression of CD13. We found varied expression of CD13 in the glioma cohorts. An increase in CD13 expression was associated with worse survival of patients. Moreover, two glioblastoma cell lines (U118, U1242) exhibited high levels of CD13 in vitro, enabling the investigation of aminopeptidase inhibition. Here, bestatin reduced proliferation, migration and colony formation of these glioblastoma cell lines, while enhancing apoptosis. In conclusion, CD13 is highly expressed by glioblastoma tissues and cell lines, and relevant for key functions of the tumor cells, indicating CD13 as a potential target in novel therapeutic strategies.

## Materials and methods

### Bioinformatic analysis

Gene expression data and corresponding clinical information of glioma patients were downloaded from The Cancer Genome Atlas (TCGA, http://cancergenome.nih.gov), and Chinese Glioma Genome Atlas (CGGA, http://www.cgga.org.cn). The CGGA encompasses two mRNA sequence databases named CGGA325 and CGGA693, containing 325 and 693 samples, respectively. Bioinformatics analyses were realized with R Language. Survival and survminer R packages were used to conduct Kaplan–Meier (K-M) survival analyses. The survival ROC package was used to generate ROC curves at 1, 3, and 5 years using the K-M method. The correlation between *CD13* expression and various clinical characteristics was plotted using beeswarm package. Univariate and multivariate Cox analysis were performed to evaluate independent prognostic factors. Gene Ontology (GO) analysis was conducted using clusterProfiler package to identify biological processes and pathways associated with the *CD13* expression. Genes related to *CD13* expression were identified using Pearson’s correlation analysis with a threshold of|r| ≥ 0.3.

### Human specimens

Brain tissue samples of 16 patients were collected during therapeutic surgical treatment from 2013 to 2014 (Department of Neurosurgery, Charité-Universitätsmedizin Berlin, Germany). Specimens of four epilepsy patients, four astrocytoma WHO °III and eight GBM patients were investigated. Neuropathologists assessed brain tissues by standard histologic markers. Characteristics of patients are shown in Supplementary Table 1, Additional File 1. Approval for the study was given by the Ethics Committee of Charité-Universitätsmedizin Berlin (application number: EA4/065/13). Investigations were performed in accordance with the obligations of scientific working with patient material. Declaration of consent was obtained from all patients.

### Immunofluorescence staining of human tissue

Following surgery, tissues were preserved in 4% paraformaldehyde (PFA) for 24 h and dehydrated in a serial dilution with rising concentrations of sucrose, subsequently. Samples were gently frozen with liquid nitrogen. Sections of 10 μm thickness were prepared. Sections were treated with Autofluorescence Eliminator Reagent (Millipore, Burlington, MA, USA) following the instructions of the manufacturer. Additionally, antigen retrieval was performed using Antigen Retrieval Reagent Universal 10x (R&D Systems) according to manufacturer's instructions. Blocking was carried out using 1% Casein/PBS + 0.1% Triton X-100 for 30 min at room temperature directly after antigen retrieval protocol. Slices were incubated with primary antibody for CD13 (rabbit, Cat #ab108310, 1/400; Abcam, Cambridge, UK) overnight at 4 °C. After several wash steps, secondary antibody was added: AlexaFluor488 anti-rabbit IgG (1/200; Dianova, Hamburg, Germany). After 1.5 h incubation at room temperature, slices were washed with PBS and water, and treated with Immunoselect Antifading Mounting Medium DAPI (Dianova).

Images were acquired by Zeiss Axio Observer Z1 fluorescence microscope (Zeiss Micro-Imaging GmbH) at room temperature. ImageJ 1.49v (NIH) was used to analyze images. Approximately 18–26 images for each patient at three different brain tissue areas were analyzed.

### Cultivation of human glioblastoma cell lines

Human glioblastoma cell lines T98G, SF188, SF767, U373, U118, SF126, and U1242 were cultured in DMEM (Thermo Fisher Scientific, Waltham, MA, USA) supplemented with 10% FBS (Gibco, Thermo Fisher Scientific, Waltham, MA, USA) and penicillin/streptomycin (100 U/mL). U87MG cells were cultured in DMEM containing 20% FBS and penicillin/streptomycin (100 U/mL). These cell lines were generously provided by Prof. Dr. Axel Ullrich from the Max Planck Institute of Biochemistry, Martinsried, Germany. The cells were maintained at 37 °C in a humidified atmosphere containing 5% CO_2_. The characteristics of these cell lines are presented in Supplementary Table 2, Additional File 2.

### Quantitative real-time PCR

To extract RNA from human glioma cells, the PureLink® RNA Mini Kit (Invitrogen, Carlsbad, CA, USA) was used. RNA concentration was measured using a plate photometer (Infinite M200, Tecan, Männedorf, Switzerland). Reverse transcription was performed using PrimeScript^™^ RT reagent Kit (Takara Bio, Kusatsu, Japan). Real-time PCR amplifications were carried out in triplicate in a 20 µL reaction volume, using the TB Green® Premix Ex Taq Kit (Takara Bio, Kusatsu, Japan) with the human *CD13* primer pairs (Fw: CATCCATCAGAGATGGCAGAC, Rev: TGCTGAAGAGATCGTTCTGG). Quantitative real-time PCR was performed using a 7900HT Fast Real-Time PCR System (Applied Biosystems, Foster City, CA, USA) to quantify the target mRNA levels, which were then normalized to * 18S* (Fw: GGCCCTGTAATTGGAATGAGTC, Rev: CCAAGATCCAACTACGAGCTT) levels. The ^ΔΔ^Ct method was used to analyze the data.

### Protein extraction and western blot

Total proteins were extracted from human glioma cells using RIPA buffer (Thermo Fisher Scientific, Waltham, MA, USA) and heated for 5 min. 4–15% Mini-PROTEAN® TGX™ Precast Gels (BIO-RAD, California, USA) were used to separate the proteins which were then transferred onto PVDF membranes. The membranes were blocked for 20 min at room temperature using StartingBlock™ (PBS) Blocking buffer (Thermo Fisher Scientific, Waltham, MA, USA) and incubated at 4 °C overnight with primary antibodies including CD13 antibody (rabbit, Cat #ab108310, 1/500; Abcam, Cambridge, UK), BAX (D2E11) (rabbit, Cat#5023, 1/1000; Cell Signaling Technology, Danvers, MA, USA ), BCL-2 (mouse, Cat#15071, 1/1000; Cell Signaling Technology), NOXA (mouse, Cat#ab13654, 1/1000; Abcam), Caspase-3 (D3R6Y) (rabbit, Cat#14220, 1/500; Cell Signaling Technology) and GAPDH antibody (mouse, Cat #ab9484, 1/1500; Abcam). After washing several times with TBS-T (0.05% Tween20), the membranes were incubated with HRP-conjugated secondary antibodies (1/200; Dianova, Hamburg, Germany) for 2 h at room temperature. The blots were washed several times before the enhanced chemo-luminescence detection kit (ECL Advance) was applied and luminescence was measured.

### Immunocytochemistry

Human glioma cells were seeded into 8-well chamber slides (ibidi, Gräfelfing, Germany) at different densities as indicated in Supplementary Table 3, Additional File 3. After 3 d of growth, cells were fixed with 4% paraformaldehyde in PBS for 20 min, followed by washing with PBS. Then, cells were blocked in 1% casein (Sigma-Aldrich, St. Louis, MO, USA) in PBS for 1 h. Cells were incubated with CD13 antibody (rabbit, Cat #ab108310, 1/400; Abcam) and phalloidin (Cat #ab176753, 1/200; Abcam) for 2 h at room temperature. After washing twice with 0.5% casein/PBS, cells were incubated with Cy3 anti-rabbit IgG (1/200; Dianova) for 2 h, protected from light. The slides were then washed with PBS and water, and the chambers were removed. Finally, the slides were mounted with DAPI-containing media (Dianova). Images were acquired at 20-fold magnification using an inverse fluorescence microscope Zeiss Axio Observer Z1 (Carl Zeiss MicroImaging GmbH, Germany).

### Flow Cytometry

All samples were measured on the BD FACSCanto II (BD Biosciences, Heidelberg, Germany) and evaluated with FlowJo software (Ashland, OR, USA).

#### Staining of CD13 (surface staining and intracellular staining)

Human glioma cells were trypsinized. For intracellular staining, the eBioscience Intracellular Fixation & Permeabilization Buffer Set (eBioscience, San Diego, CA) was used according to the manufacturer's instructions. Briefly, cells were fixed and permeabilized before being incubated with PE anti-human CD13 Antibody (mouse, Cat #310704, 1/20; BioLegend, London, UK) in Perm Buffer for 30 min at room temperature. For surface staining, cells were directly incubated with the PE anti-human CD13 Antibody in 0.5% BSA/PBS for 15 min on ice. After staining, cells were washed and resuspended in 0.5% BSA/PBS. DAPI was added before measurement to exclude dead cells.

#### Annexin-V staining

Human glioma cells were treated with 500 µg/mL bestatin (Cayman, Neratovice, Czech) or without drug for 48 h. After trypsinization and collection, 1 × 10^5^ cells/sample were used for Annexin-V staining. Following two washes with PBS, cells were incubated with FITC Annexin V (mouse, Cat #640906, 1/20; BioLegend, London, UK) and DAPI in Annexin V Binding Buffer (BioLegend) for 15 min at RT protected from light.

#### Detection of reactive oxygen species (ROS)

Human glioma cells were treated with 500 µg/mL bestatin or without drug for 48 h. After trypsinization and collection, 1 × 10^5^ cells/sample were used for ROS detection. CellROX™ Deep Red Flow Cytometry Assay Kit (Thermo Fisher Scientific, Waltham, MA, USA) was utilized according to the manufacturer's protocol. Briefly, CellROX® Deep Red Reagent (50mM) was incubated with cells at 37 °C for 1 h to detect ROS production.

### Proliferation assay

Human glioma cells were seeded into a 96-well plate in a no-phenol red medium at various densities as indicated in Supplementary Table 3, Additional File 3. After 24 and 48 h of growth, the cells were treated with different concentrations of bestatin (0, 62.5, 125, 250, 500 µg/mL) for 24 and 48 h. The MTT Cell Proliferation Assay Kit (Cayman, Neratovice, Czech) was utilized according to the manufacturer's instructions. The absorbance was measured at 570 nm using a microplate reader (Infinite M200, Tecan, Männedorf, Switzerland). The experiment was replicated twice, with triplicate wells for each replication.

### Scratch assay

Human glioma cells were collected and adjusted to different cell numbers as indicated in Supplementary Table 3, Additional File 3. A total of 70 µl of cells was added into the insert well of a Culture-Insert 2 Well 24 plate (ibidi, Gräfelfing, Germany), while 340 µl of cells was added around the insert. After 24 h of growth, cells were starved for up to 12 h in DMEM without supplements. The inserts were removed, and residual cells were washed away with PBS. Next, cells were treated with 250 µg/mL bestatin or without drug, and pictures were taken at 0 h, 12 h, and 24 h with 10x magnification. The scratch area was calculated using ImageJ software (National Institutes of Health, Bethesda, MD, USA).

### Colony formation assay

Human glioma cells were seeded into 6-well plates (250 cells/well). After 24 h of growth, cells were treated with 250 µg/mL bestatin or without drug and cultured for approximately two weeks. Following washing with PBS, cells were fixed with methanol for two minutes and then stained with 2.3% crystal violet solution (Sigma-Aldrich, St. Louis, MO, USA) for 15 min. After several washes with distilled water, pictures were taken, and the number of colonies was counted using a microscope. A cell population with more than 50 cells was defined as a colony.

### CD13 activity assay

Human glioma cells were treated with 500 µg/mL bestatin or without drug for 48 h. Cells were trypsinized and collected, and 1 × 10^6^ cells/sample were used for CD13 activity assay. The Aminopeptidase N (APN/CD13) Activity Assay Kit (Fluorometric) (Abcam, Cambridge, UK) was utilized according to the manufacturer's protocol. A standard curve was constructed using AFC Standard. Samples were incubated with the substrate, and the fluorescence (Ex/Em = 384/502 nm) was measured in kinetic mode for 60 min at 37 °C using a microplate reader (Infinite M200, Tecan). Results were calculated as µU/mL of substrate cleaved.

### Statistics

Statistical analyses were performed using GraphPad Prism 8 (San Diego, CA, USA), and data are presented as mean ± SD. Comparisons between groups were analyzed using two-tailed unpaired Student’s t-tests or one-way ANOVA with Bonferroni correction as indicated. A P-value < 0.05 was considered statistically significant.

## Results

### High CD13 expression correlates with poor glioma patient survival and increased malignancy

**Fig. 1:**
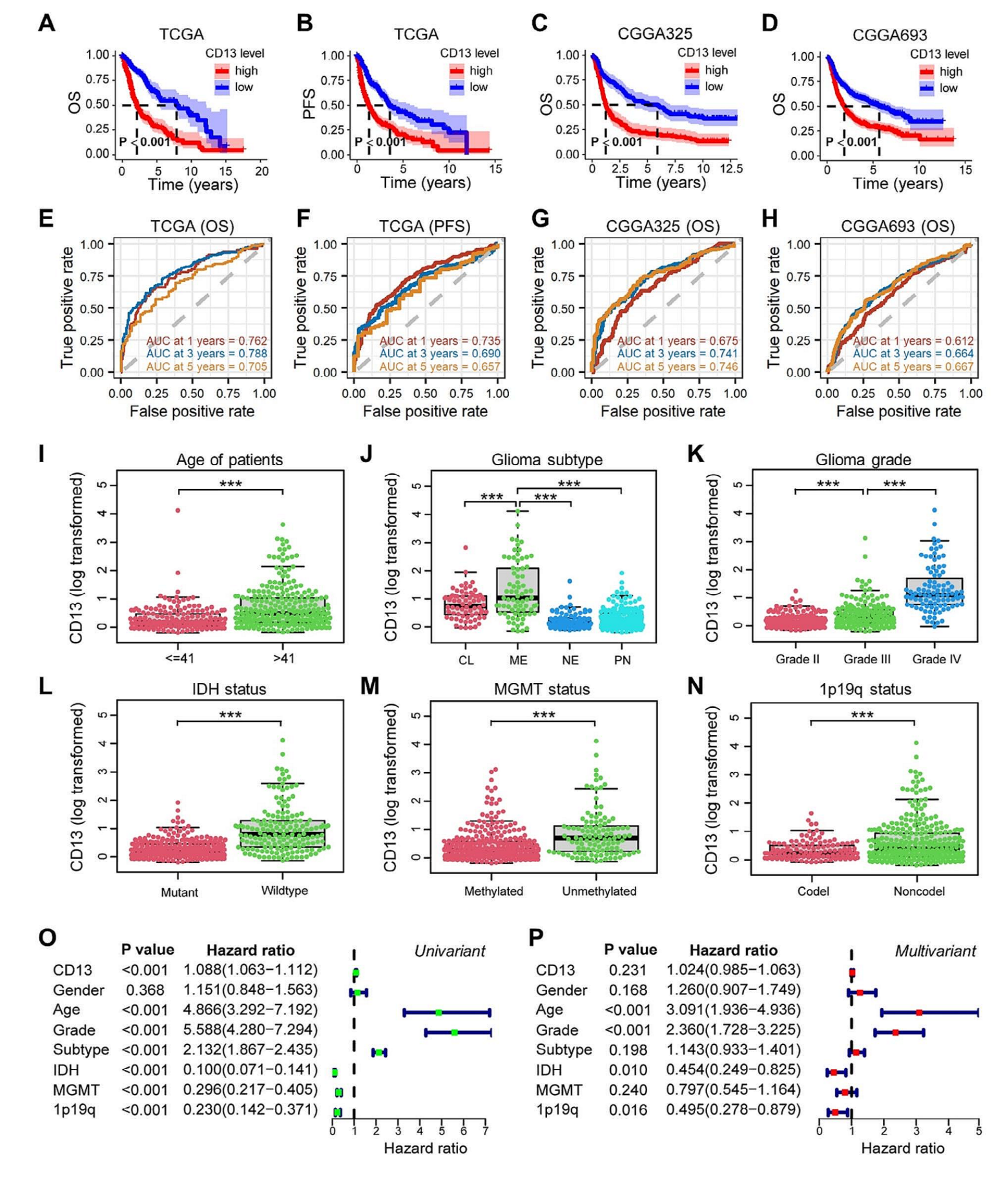
Prognostic value of *CD13* expression for glioma patients. **(A-D)** Kaplan-Meier curves for overall survival (OS) (*n* = 666; **A**) and for progression-free survival (PFS) (*n* = 670; **B**) of glioma patients determined by TCGA database. Kaplan-Meier curves for OS ascertained by CGGA325 database (*n* = 313; **C**) and CGGA693 database (*n* = 657; **D**). **(E-H)** ROC (receiver operating characteristic) curves for *CD13* expression as a prognostic marker of overall survival (*n* = 666; **E**) and PFS (*n* = 670; **F**) generated by TCGA database. ROC curves for *CD13* expression as a prognostic marker of overall survival determined by CGGA325 database (*n* = 313; **G**) and CGGA693 database (*n* = 657; **H**). **(I-N)** *CD13* expression classified by age of patients **(I)**, glioma subtype (**J**; CL: classical; ME: mesenchymal; NE: neural; PN: proneural), glioma grade **(K)**, *IDH* mutation status **(L)**, *MGMT* methylation status **(M)**, and 1p19q codeletion status **(N)** ascertained by TCGA database (*n* = 450). **(O,P)** Univariate **(O)** and multivariate **(P)** Cox regression analyses of *CD13* expression (*n* = 447; analyzed data of TCGA database). ***P < 0.001 (I, L-N, unpaired Student´s t test; J,K, ANOVA and Bonferroni correction)

First, we were interested in the impact of *CD13* gene expression on survival of glioma patients, including low-grade glioma (LGG, grade II) and high-grade glioma (HGG, grade III and IV). Here, we analyzed different databases to collect information. The Kaplan-Meier survival analyses of The Cancer Genome Atlas (TCGA) and Chinese Glioma Genome Atlases (CGGA325 and CGGA693) revealed that high *CD13* expression is associated with a significantly shorter survival of glioma patients. Using data of the TCGA database, the median survival times for patients with low and high *CD13* expression were 7.88 years (95% CI: 5.30–12.18 years) and 2.12 years (95% CI: 1.78–2.63 years), respectively (Fig. 1A). Additionally, this database revealed that high *CD13* expression is associated with a shorter progression-free survival. Here, the median survival times for patients with low *CD13* expression was 3.58 years (95% CI: 3.17–6.21 years) and 1.30 years (95% CI: 1.09–1.60 years) for *CD13* high expression (Fig. 1B). The observed differences in the TCGA database between *CD13* low and *CD13* high expressing patients could be confirmed by the other databases.

In the CGGA325 database, the median survival times for patients with low and high *CD13* expression were 5.90 years (95% CI: 3.46–7.05 years) and 1.23 years (95% CI: 1.04–1.65 years), respectively (Fig. 1C). Similarly, in the CGGA693 database, the median survival times for patients with low *CD13* expression were 5.68 years (95% CI: 4.57–7.40 years) and with high *CD13* expression were 1.87 years (95% CI: 1.56–2.21 years; Fig. 1D). To evaluate the prognostic value of *CD13*, we performed receiver operating characteristic (ROC) curve analysis. Most of area under the curve (AUC) values for *CD13* at 1, 3, and 5 years were around or greater than 0.7 (Fig. 1E-H), which means that *CD13* could be a relevant prognostic marker for glioma. Moreover, the TCGA database analysis showed that *CD13* expression is related to different features of glioma (Fig. 1I-N). *CD13* expression is higher in older patients (Fig. 1I) and in tissues of the mesenchymal subtype (Fig. 1J). Furthermore, the expression increased with the grade of glioma (Fig. 1K) and is correlated with *IDH* wildtype (Fig. 1L), *MGMT* unmethylated status (Fig. 1M), and 1p19q non-codeletion (Fig. 1N). Univariate Cox analysis of survival data indicated that *CD13* expression is a high-risk factor for glioma patients like age, grade, and subtype, while *IDH* mutation, *MGMT* methylation, and 1p19q codeletion were low-risk factors (Fig. 1O). However, multivariate Cox analysis (Fig. 1P) indicated that only age and grade of malignancy are high-risk factors.

Analyses of databases revealed that CD13 could be a potential prognostic marker for glioma patients and that high *CD13* gene expression indicates poor survival and high malignancy.

### CD13 gene expression is highly associated with extracellular matrix signaling pathways in gliomas

**Fig. 2:**
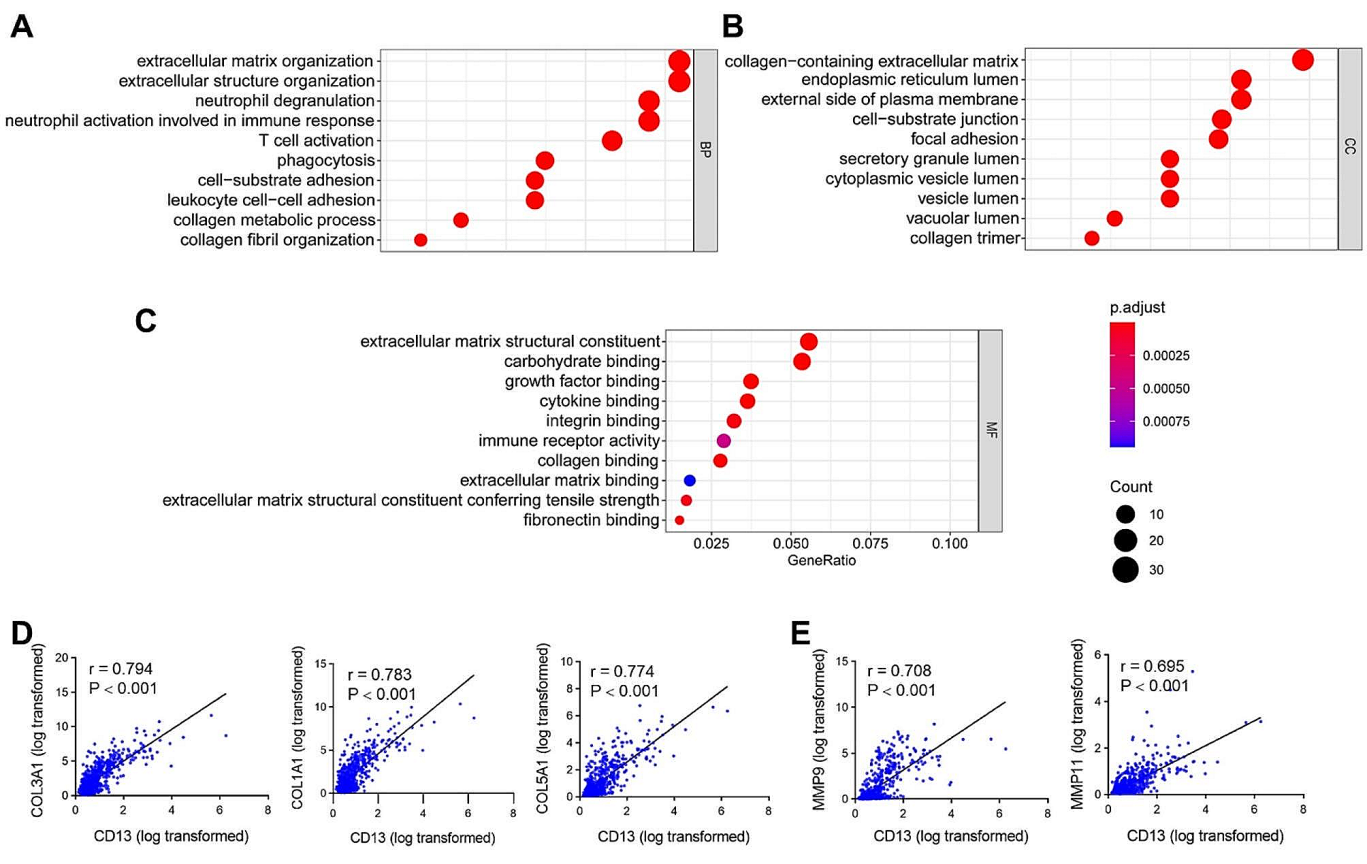
Gene Ontology enrichment for CD13 co-expressed genes in human glioma samples. **(A-C)** The top 10 enriched biological process (BP; **A**), cellular component (CC; **B**), and molecular function (MF; **C**) categories for the co-expressed genes of *CD13* obtained by TCGA database. The size of the bubbles indicates the number of genes related to the pathway, and the color indicates the level of significance of the enrichment. The GeneRatio value specifies the total number of genes annotated in the pathway. **(D,E)** Pearson’s correlation analysis showed that *CD13* is positively correlated with several collagens (COL; **D**) and matrix metalloproteinases (MMP; **E**) (*n* = 672). The correlation coefficients (r) and associated P-values are shown

Identifying co-expressed genes can provide valuable insights into potential functions of CD13 in glioma. In this study, we used Pearson’s correlation analysis to predict co-expressed genes of *CD13* by the TCGA database. With a correlation coefficient of|r| ≥ 0.3, a total of 1514 co-expressed genes were obtained (Supplementary Table 4, Additional File 4). To investigate the functions of these co-expressed genes, we conducted functional analysis using the Enrichr database, including three categories of Gene Ontology (GO) functional annotation: biological process (BP), cellular component (CC), and molecular function (MF). The enriched GO functions for these co-expressed genes included extracellular matrix organization, extracellular structure organization, cell-substrate adhesion, collagen metabolic process, and collagen fibril organization in the BP category (Fig. 2A). In the CC category, cell-substrate junction, focal adhesion, and collagen trimer functions were found (Fig. 2B). Similar functions were also observed in the MF category (Fig. 2C). Especially, based on Pearson’s correlation analysis, we found a strong correlation between *CD13* and collagens (Fig. 2D) as well as matrix metalloproteinases (Fig. 2E, Supplementary Table 5, Additional File 5).

These results suggest that CD13 plays a crucial role in extracellular matrix signaling pathways, which may contribute to cell adhesion and migration in glioma.

### CD13 expression is increased on RNA and protein level of glioblastoma patient tissues

**Fig. 3:**
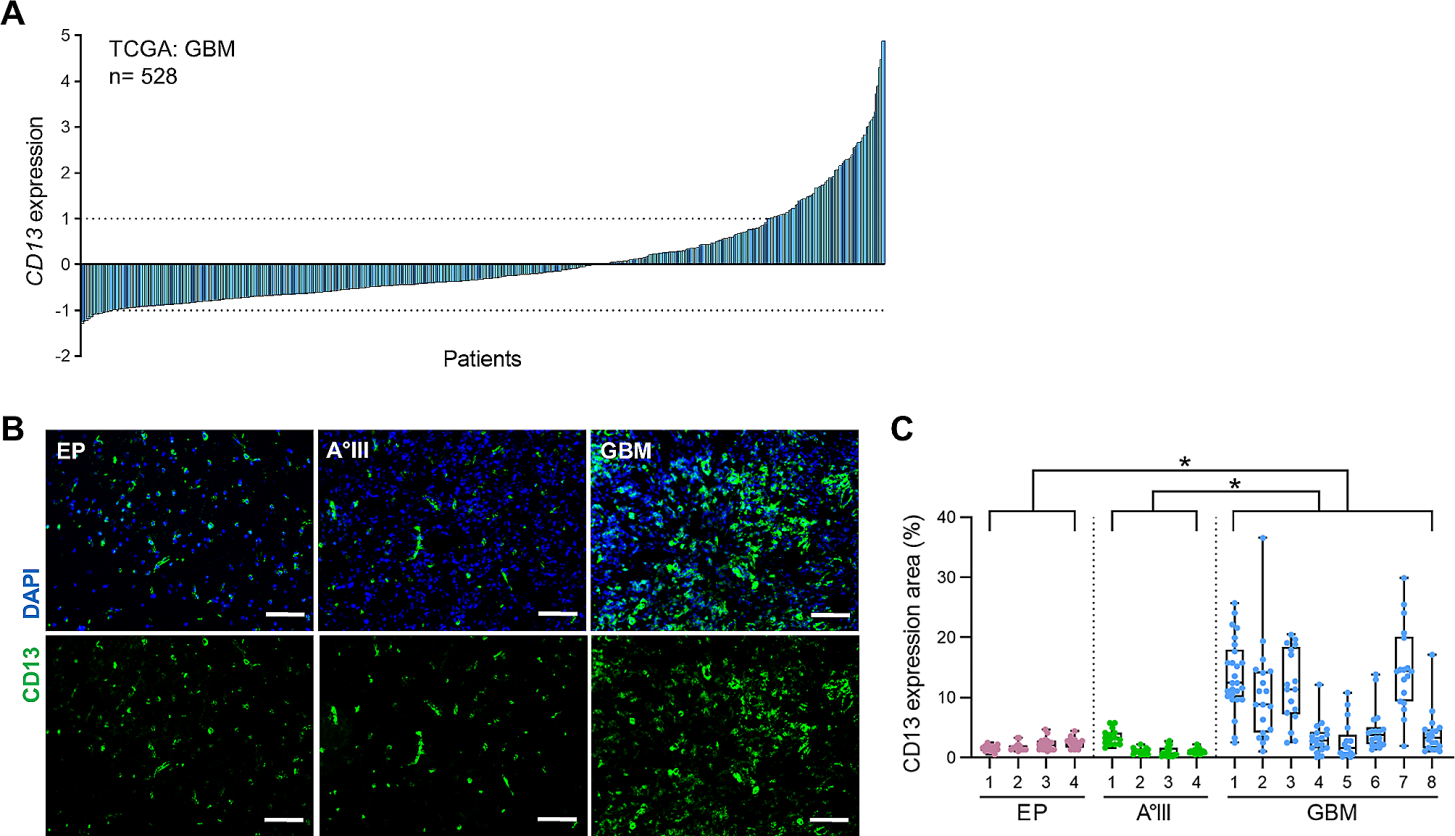
Tissues of glioblastoma patients express CD13 on RNA and protein level. **(A)** TCGA database was analyzed concerning *CD13* gene expression of glioblastoma tissues. Relative gene expression of *CD13* is depicted of each patient (Firehose Legacy, mRNA data, U133 microarray, *n* = 528). Dotted line: relative gene expression “1” (upregulated) and “-1” (downregulated). **(B)** Brain tissues of patients suffered from epilepsy (EP), astrocytoma grade III (A°III) and glioblastoma (GBM) were stained for CD13 (*green*) and nuclei (DAPI, *blue*). Scale bars, 100 μm. **(C)** Graph depicts area of CD13 expression within tissue of different brain pathologies (*n* = 4–8, each dot represents data of one image, three different tissue regions and 18–26 images/patient). *P < 0.05 (ANOVA and Bonferroni correction)

Based on the fact, that a higher *CD13* gene expression was associated with the grade of malignancy in glioma, we analyzed single data of glioblastoma patients (glioma grade IV) from TCGA database. Here, we found various expression of *CD13* (Fig. 3A). Besides the gene expression, we investigated the protein expression of CD13 of glioblastoma tissues in comparison to astrocytoma grade III (A°III) and epilepsy patient (EP) specimens. Tissues were collected directly after surgical resection. Processed brain tissues were stained for CD13 (Fig. 3B) and analyzed concerning the area of CD13 expression. All analyzed tissues showed CD13 staining, whereby CD13 expression increased with grade of glioma. Interestingly, we found a widespread distribution of CD13 in GBM specimens (Fig. 3C).

Thus, we confirmed that CD13 is highly expressed in GBM but to varying degrees.

### Human glioblastoma cell lines express CD13 to various extent

**Fig. 4:**
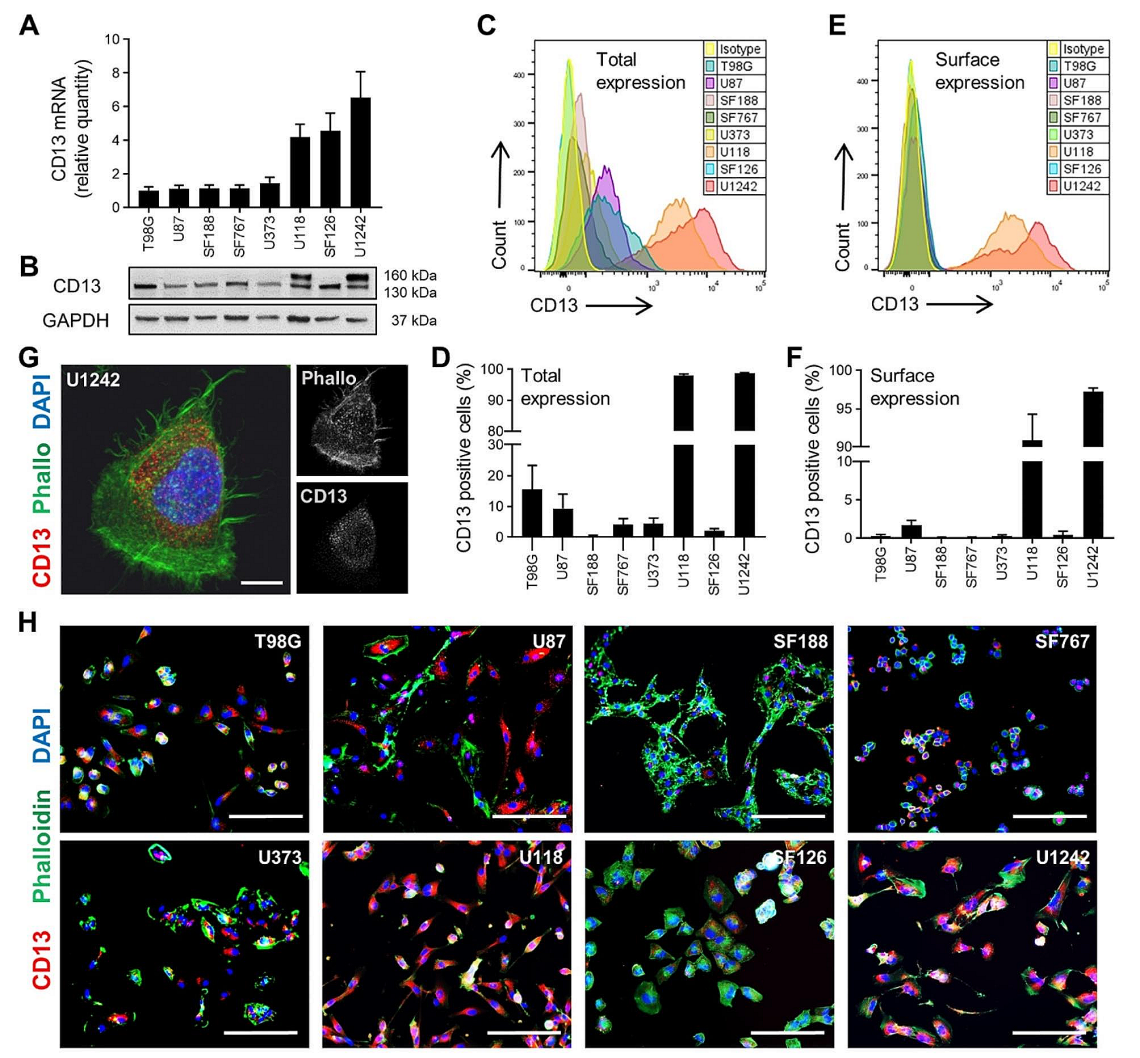
The expression level and localization of CD13 of eight different human glioblastoma cell lines. **(A)** Quantitative analysis of *CD13* mRNA expression using qRT-PCR (*n* = 3, independent cell preparations). **(B)** An example of western blot detecting intracellular (130 kDa) and surface (160 kDa) CD13 expression (*n* = 2, independent cell preparations). GAPDH (37 kDa) was used as control. Full length blots are presented in Additional file 7. **(C-F)** Flow cytometric analyses of total **(C,D)** and surface **(E,F)** expression of CD13. Representative histograms show total **(C)** and surface **(E)** expression of CD13. Quantitative analysis of the percentage of cells expressing CD13 in total **(D)** or on surface **(F)** (*n* = 4, independent cell preparations). **(G,H)** Immunofluorescence staining of glioblastoma cell lines: CD13 (*red*), F-actin (phalloidin, *green*) and nuclei (DAPI, *blue*). Confocal image of U1242 cell showing the dot-like distribution of CD13. Scale bar, 30 μm **(G)**. Immunofluorescence images of eight different cell lines. Scale bars, 400 μm **(H)**

To evaluate in detail the impact of CD13 on glioma cells, we analyzed eight different glioblastoma cell lines concerning their mRNA and protein level of CD13. Results obtained from qRT-PCR indicated that T98G had the lowest *CD13* mRNA expression, whereas U1242 had the highest *CD13* mRNA expression. Most of the analyzed cell lines (U87MG, SF188, SF767 and U373) showed only low gene expression of *CD13*, while U118 and SF126 revealed higher expression (Fig. 4A). Protein expression levels were analyzed by western blot (Fig. 4B), flow cytometry (Fig. 4C-F), and immunocytochemistry (Fig. 4G,H). The data demonstrated that CD13 protein was expressed by all cell lines. Analyses of SF188 and SF126 by various methods revealed lowest protein expression of CD13. In contrast, U118 and U1242 showed the highest protein expression. Interestingly, only these cell lines depicted two bands in western blot (Fig. 4B) and highly express CD13 on their cell surface (Fig. 4E,F) detected by flow cytometry. However, confocal imaging revealed a rather cytoplasmic expression of CD13 in U1242 cells (Fig. 4G).

These findings suggest that the expression levels and localization of CD13 varied among different glioblastoma cell lines.

### Inhibition of CD13 reduced proliferation, migration and colony formation of human glioblastoma cell lines dependent on the degree of CD13 expression

To investigate the function of CD13 on glioblastoma cell lines, bestatin was used to inhibit the aminopeptidase. We analyzed, the impact of bestatin on various glioblastoma cell lines concerning their proliferation, migration and colony formation. For these in vitro assays, we selected representative cell lines based on their CD13 expression level. U118 and U1242 were chosen as high-expression CD13 cell lines (CD13^h^), T98G as a medium-expression CD13 cell line (CD13^m^), and SF188 as a low-expression CD13 cell line (CD13^l^).

**Fig. 5:**
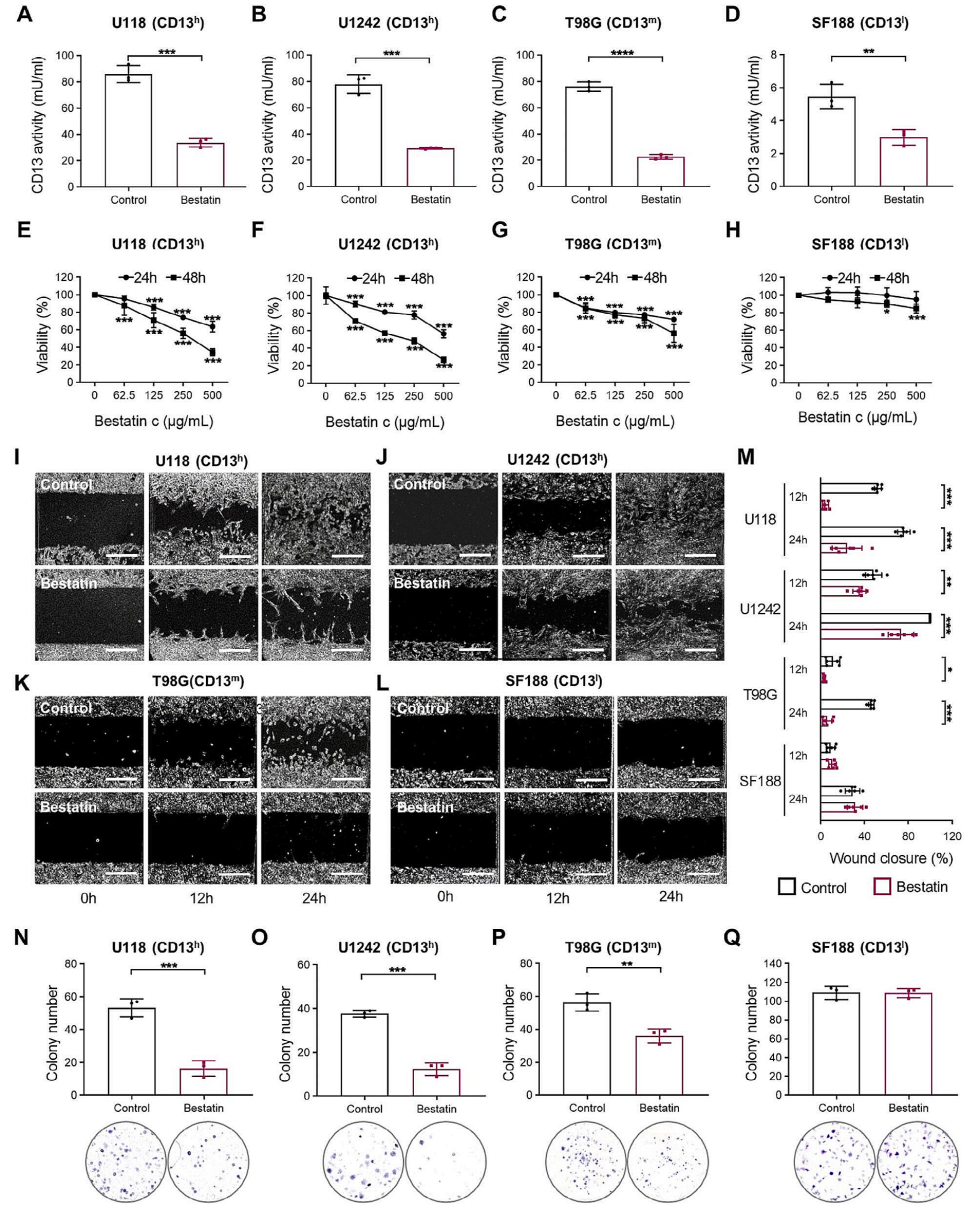
The impact of bestatin on cell function in human glioblastoma cell lines. **(A-D)** Quantitative analysis of CD13 activity following bestatin treatment (500 µg/mL) is depicted (*n* = 3, independent cell preparations). **(E-H)** Cell growth curves at 24 and 48 h after treatment with 0, 62.5, 125, 250, 500 µg/mL bestatin (*n* = 6 wells/condition of two independent experiments). Statistics relative to 0 h. **(I-L)** Representative images of the cell scratch assay at starting point (0 h), at 12 and 24 h. Scale bars, 400 μm. **(M)** Quantification of the percentage of wound closure at 12 and 24 h (250 µg/mL bestatin). (*n* = 6 wells/condition of two independent experiments). **(N-Q)** Quantification and representative images of colony formation following bestatin treatment (250 µg/mL) are depicted (*n* = 3 wells/condition; one representative experiment of three independent experiments is shown). *P < 0.05, **P < 0.01, ***P < 0.001 (unpaired Student’s t-test). CD13^h^, high CD13 expression; CD13^m^, medium CD13 expression; CD13^l^, low CD13 expression

Besides expression, also activity of CD13 is relevant for aminopeptidase function [37]. Interestingly, the high and intermediate CD13 expressing cell lines revealed a comparable level of CD13 activity (Fig. 5A-C) but SF188 showed very low levels (Fig. 5D). However, administration of bestatin significantly reduced the CD13 activity in all cell lines (Fig. 5A-D). The proliferation assay demonstrated that bestatin reduced cell viability of U118 and U1242 in a time and concentration-dependent manner (Fig. 5E,F), while the effect was less pronounced in T98G cells (Fig. 5G). Proliferation of SF188 cell line was only affected by high concentrations of bestatin at 48 h of treatment (Fig. 5H). The scratch assay indicated that bestatin inhibited cell migration of U118, U1242, and T98G, but not SF188 cell line (Fig. 5I-M). Additionally, we analyzed the capability of cells to build colonies from single cells. Here, we found that bestatin significantly reduced colony formation of U118 and U1242 cells (Fig. 5N,O), while the effect was less pronounced in the T98G cell line (Fig. 5P) and absent in SF188 cells (Fig. 5Q).

These results suggest that bestatin inhibits cell proliferation, migration, and colony formation in a CD13-dependent manner.

### Inhibition of CD13 induces apoptosis in human glioblastoma cell lines

**Fig. 6:**
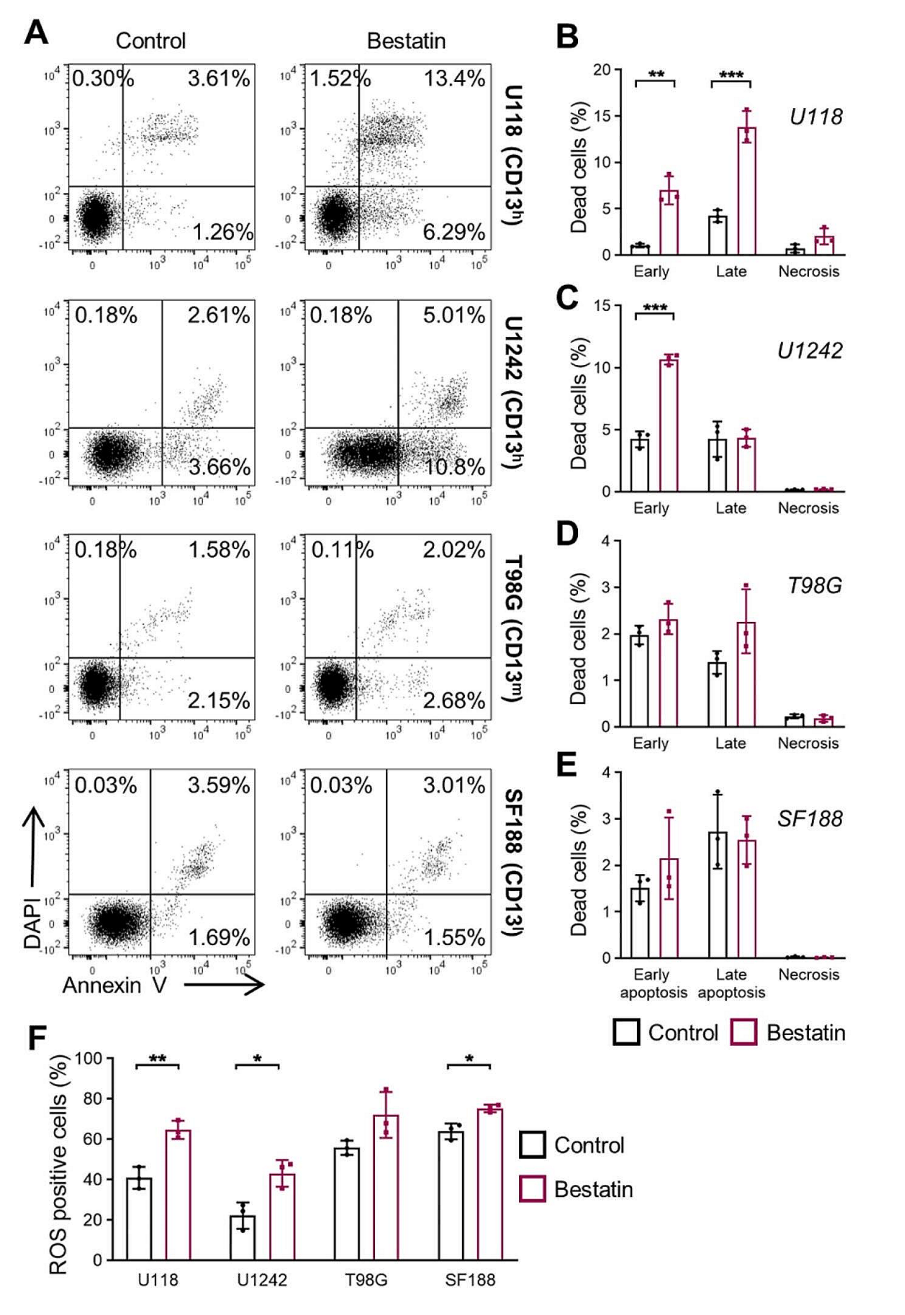
The impact of CD13 on apoptosis in human glioblastoma cell lines and potential mechanism. **(A)** Representative dot plots of Annexin V staining. Following 48 h of bestatin treatment (500 µg/mL), cells were trypsinized, and cells were stained with DAPI and Annexin V (FITC). Early apoptotic cells (Annexin V^+^DAPI^-^), late apoptotic cells (Annexin V^+^DAPI^+^), necrotic cells (Annexin V^-^DAPI^+^). **(B-E)** Quantitative analysis of the percentage of early apoptotic, late apoptotic, and necrotic cells (*n* = 3, independent cell preparations). **(F)** Quantitative analysis of ROS positive cells (*n* = 3, independent cell preparations). After 500 µg/mL bestatin treatment for 48 h, cells were trypsinized, and cell suspension was incubated with CellROX^®^ Detection Reagent. *P < 0.05, **P < 0.01, ***P < 0.001 (unpaired Student’s t-test). CD13^h^, high CD13 expression; CD13^m^, medium CD13 expression; CD13^l^, low CD13 expression

Bestatin can act by induction of apoptosis [38–40]. Apoptosis is a highly regulated process that can be divided into early and late stages based on distinct cellular changes and molecular events. Detecting both early and late events of the apoptosis cascade is important for a comprehensive understanding of the apoptotic process and to evaluate the effectiveness of therapeutic interventions [41]. Therefore, we investigated the apoptotic pathway of the four human glioblastoma cell lines via Annexin V staining (Fig. 6A). The results showed that bestatin increased both early and late apoptosis of U118 cells (Fig. 6B), while in U1242 cells, it only enhanced early apoptosis (Fig. 6C), and no effect on apoptosis was observed in T98G and SF188 cells (Fig. 6D,E). Necrosis was low in all cell lines and not affected by bestatin treatment. Furthermore, we analyzed the expression levels of various proteins, including pro-apoptotic markers like BAX and NOXA, the anti-apoptotic molecule BCL-2 as well as Pro and Cleaved Caspase-3 (CAS-3) using western blotting (Supplementary Fig. 1, Additional File 6). Our results indicated that bestatin treatment upregulated the expression of BAX, NOXA, and Cleaved Caspase-3, but did not affect the expression of BCL-2 and Pro Caspase-3 in U118 and U1242 cells. In T98G cells, there was no obvious change in the expression of these proteins upon addition of bestatin. In SF188 cell line we could not detect expression of BAX, NOXA, and BCL-2, and no changes in the expression of Pro and Cleaved Caspase-3 following bestatin treatment. To further investigate the underlying mechanism, we assessed the level of reactive oxygen species (ROS) production (Supplementary Fig. 2, Additional File 6) that is relevant for the damage to proteins, nucleic acids, lipids, membranes and organelles, which can lead to activation of cell death processes such as apoptosis [42]. Our findings revealed that administration of bestatin led to a significant increase in ROS levels in U118, U1242 cells, while no effect was observed in T98G cells (Fig. 6F). The effect of bestatin on ROS production of SF188 cells was less pronounced than in the CD13 high expressing cell lines.

Therefore, our study suggests that bestatin induces cell apoptosis through the ROS-activated intrinsic pathway in a CD13-dependent manner.

## Discussion

In our present study, we investigated the role of CD13 as a prognostic marker in glioma and explored its potential as a therapeutic target. Our findings demonstrated that high expression of CD13 was associated with poor prognosis of glioma patients. Moreover, we found that bestatin exhibited significant inhibitory effects on various cellular processes of glioblastoma cells, including cell proliferation, migration, colony formation, and induction of cell apoptosis. Remarkably, these effects were dependent on the expression level of CD13.

CD13 is a protein that is frequently overexpressed in various types of cancers, promoting cancer progression and contributing to chemotherapy resistance in many cases [43–46]. However, it is important to note that there are some contrary findings in the literature regarding CD13 expression in specific cancer types. For instance, Mawrin et al. reported reduced CD13 expression in high-grade meningiomas [47], and Marletta et al. found that low CD13 expression is associated with a worse outcome in meningiomas [48]. In addition, Gao et al. demonstrated that low CD13 expression in patients with non-small cell lung cancer is associated with poor survival [49]. Although CD13 has been studied in various cancers, limited research has specifically focused on its role in GBM. In our study, we investigated CD13 expression in GBM and found that CD13 is also overexpressed in this aggressive form of brain tumors. This expands our understanding of the involvement of CD13 in GBM and highlights its potential significance as a target for therapeutic interventions in this particular cancer type.

The prognostic value of CD13 in glioma was assessed using survival analysis, ROC curve and Cox analysis. Our analysis of the TCGA, CGGA325, and CGGA693 datasets revealed that higher CD13 expression is predictive of unfavorable OS. Moreover, within the TCGA dataset, high CD13 expression was associated with diminished PFS. The absence of PFS calculation for CGGA325 and CGGA693 is attributed to the lack of this parameter within these datasets. The observed variability in OS values is principally driven by disparities in patient demographics and methodologies for data processing. However, in all datasets we observed the same tendency for the impact of CD13 expression on glioma patient survival. The AUC values for CD13 were predominantly above 0.7, indicating that CD13 could serve as a reliable prognostic marker for glioma. Univariate Cox analysis revealed that CD13 expression is a significant high-risk factor for glioma patients. However, when conducting multivariate analyses, the significance of CD13 expression was not observed. The lack of significance in the multivariate analysis regarding CD13 expression suggests that other variables included in the analysis, such as age, tumor grade, or subtype, may have confounded the association. These variables might have a stronger influence on patient outcomes compared to CD13 expression, thereby masking its independent prognostic significance.

To evaluate the expression pattern of CD13 in different human glioblastoma cell lines, we utilized various detection methods. We observed slight differences in CD13 expression levels when several techniques were applied. This variability could be attributed to the sensitivity of the detection methods used or the presence of discrete glycoforms or conformations of the CD13 protein that might not be recognized by certain reagents [15]. Furthermore, our investigation revealed the presence of two different isoforms of CD13: intracellular CD13 (130 kDa) and surface CD13 (160 kDa), which is consistent with previous literature on the subject [8, 15]. CD13 expression among different human glioblastoma cell lines and within glioblastoma tissues suggests that CD13 expression showed a high variation between individuals. Such heterogeneity in CD13 expression may have implications for treatment response, as cells with lower CD13 expression could potentially be less susceptible to therapeutic interventions. Considering these findings, personalized treatment strategies that take into account individual characteristics and profiles related to CD13 expression or activity could be beneficial. By tailoring treatment approaches based on the specific CD13 expression levels in each patient may enhance the efficacy of therapies and improve patient outcomes.

We observed that bestatin affected the activity of CD13 in all tested cell lines, indicating its effectiveness as an inhibitor. However, the impact of CD13 inhibition on cellular function appeared to be more dependent on the expression levels of CD13. Cells with higher CD13 expression are more likely to rely on CD13 activity for carrying out specific functions. Consequently, when CD13 is inhibited by bestatin, the impact on these functions is expected to be more pronounced in cell lines with higher CD13 expression levels. In contrast, cells with lower CD13 expression may possess compensatory mechanisms or alternative pathways that can partially compensate for the loss of CD13 activity. In such cases, the effects of CD13 inhibition by bestatin may be attenuated or less apparent. It should be mentioned that bestatin can also act on other aminopeptidases like Arginyl-Aminopeptidase (RNPEP), Leucyl- and Cystinyl-Aminopeptidase (LNPEP) and Leukotriene A-4 Hydrolase (LTA4H) besides CD13 [50, 51]. Thus, some of the observed effects could be amplified by inhibition of these aminopeptidases. To exclude potential unspecific influences of bestatin, future experiments should include the knockout of *CD13* in glioblastoma cells to define the exclusive role of CD13 more in detail. Glioblastoma is a highly aggressive form of brain cancer known for its invasive nature within the brain. While glioblastoma is generally not considered metastatic tumor in the traditional sense. Reported metastatic rates varied from as low as 0.2% to as high as 2% [52], meaning they do not typically spread to distant organs outside the central nervous system. However, inhibition of the cell migration and invasion could still be valuable in limiting the local spread and invasive behavior of the tumor within the brain. In this context, our in vitro study indicated that bestatin inhibits cell migration. Gene Ontology enrichment further revealed that CD13 is implicated in matrix signaling pathways, which may contribute to cell adhesion and migration. Therefore, targeting CD13 with an inhibitor like bestatin may potentially disrupt tumor invasion and migration, which could have therapeutic implications for glioblastoma treatment.

Bestatin has been reported to possess antioxidant properties and can scavenge ROS [37, 38]. ROS accumulation can lead to oxidative stress and cell damage, thereby triggering apoptosis. It has been shown that bestatin that acts through the CD13 enzyme, increases intracellular ROS levels, and induces cell apoptosis in some cancers [37, 53]. However, the role of ROS in bestatin-induced cell death in glioma cells remains unknown. It has been reported that ROS can upregulate the expression of BAX, a pro-apoptotic protein, and promote its translocation to the mitochondria [54]. The insertion of BAX into the mitochondrial outer membrane results in mitochondrial outer membrane permeabilization (MOMP), leading to the release of apoptotic factors such as cytochrome c into the cytosol. This event activates downstream signaling pathways, including the activation of Caspase-3. Caspase-3, once activated, triggers a cascade of proteolytic events that ultimately execute apoptosis. NOXA, another critical player in apoptosis, functions by neutralizing the anti-apoptotic function of BCL-2 family members, thereby preventing their interaction with pro-apoptotic proteins like BAX or BAK. This disruption allows BAX and BAK to promote MOMP, leading to the activation of the apoptotic pathway. Consistent with previous studies, our results demonstrated that treatment with bestatin increases ROS generation in human glioma cells. This ROS upregulation subsequently leads to the upregulation of pro-apoptotic proteins, such as BAX and NOXA, and the activation of the apoptotic pathway, ultimately resulting in cell apoptosis. Interestingly, the anti-apoptotic protein BCL-2 was not affected by bestatin treatment, suggesting the presence of an alternative pathway.

## Conclusions

In conclusion, our study provides valuable insights into the role of CD13 as a prognostic marker and therapeutic target in glioma. However, it is important to note that further research and preclinical and clinical studies are needed to assess the efficacy and safety of such interventions in glioblastoma patients.

### Electronic supplementary material

Below is the link to the electronic supplementary material.

Supplementary Material 1

Supplementary Material 2

Supplementary Material 3

Supplementary Material 4

Supplementary Material 5

Supplementary Material 6

Supplementary Material 7

## Data Availability

The datasets used and analyzed during the current study are available from the corresponding author (P.V.) on reasonable request.

## Abbreviations

GBM: Glioblastoma
APN: Aminopeptidase N
TAMs: Microglia/macrophages
TCGA: The Cancer Genome Atlas
CGGA: Chinese Glioma Genome Atlas
GO: Gene Ontology
PFA: Paraformaldehyde
LGG: Low-grade glioma
HGG: High-grade glioma
OS: Overall survival
PFS: Progression-free survival
ROC: Receiver operating characteristic
CL: Classical
ME: Mesenchymal
NE: Neural
PN: Proneural
BP: Biological process
CC: Cellular component
MF: Molecular function
COL: Collagen
MMP: Matrix metalloproteinases
EP: Epilepsy
CAS-3: Caspase-3
ROS: Reactive oxygen species
RNPEP: Arginyl-Aminopeptidase
LNPEP: Leucyl- and Cystinyl-Aminopeptidase
LTA4H: Leukotriene A-4 Hydrolase
MOMP: Mitochondrial outer membrane permeabilization
BAX: Bcl-2-associated X protein
BCL-2: B-cell lymphoma 2
NOXA: Phorbol-12-myristate-13-acetate-induced protein 1
GAPDH: Glyceraldehyde 3-phosphate dehydrogenase
DAPI: 4’,6-diamidino-2-phenylindole
