# Supplementary material for: CD13 expression affects glioma patient survival and influences key functions of human glioblastoma cell lines in vitro
Source: BMC Cancer. 2024 Mar 22;24:369. doi: 10.1186/s12885-024-12113-z (PMC10960415; doi:10.1186/s12885-024-12113-z)
Supplement: Supplementary file 1 — Supplementary Material 1 [file 12885_2024_12113_MOESM1_ESM.pdf]

Supplementary Table 1. Characteristics of patients

	Value
**Age (mean ± SD)**	**50.63 ± 15.97**
GBM	57.88 ±18.20
A°III	39.75 ±8.380
EP	47.00 ± 10.80
**Gender (f/m)**	**10/6**
GBM	5/3
A°III	3/1
EP	2/2
**Localisation of tumor**	**GBM/A°III**
frontal	1/1
temporal	3/2
occipital	2/0
insula	0/1
operculum	2/0
**MGMT Status**	**methylated/unmethylated**
GBM	3/5
**IDH Status**	**wildtype/mutant/na**
GBM	5/1/2
A°III	1/2/1
**Recurrence**	**primary/relapse**
GBM	6/2

GBM, glioblastoma; A° III, anaplastic astrocytoma (WHO grade III); EP, epilepsy; f, female; m, male; MGMT, O-6-methylguanin-DNA-methyltransferase; IDH, isocitrate dehydrogenase; na, not assigned.
