# Supplementary material for: CD13 expression affects glioma patient survival and influences key functions of human glioblastoma cell lines in vitro
Source: BMC Cancer. 2024 Mar 22;24:369. doi: 10.1186/s12885-024-12113-z (PMC10960415; doi:10.1186/s12885-024-12113-z)
Supplement: Supplementary file 2 — Supplementary Material 2 [file 12885_2024_12113_MOESM2_ESM.pdf]

Supplementary Table 2. Characteristics of human glioma cell lines

Cell line	Origin	Disease	Morphology	Features	Reference
T98G	Male, 61-year old	Glioblastoma	Fibroblast	Karyotype: hyperpentaploid<br>Doubling time: 24-28h	Stein GH et al. [1]
U87MG	Male, 44-year old	Glioblastoma	Epithelial	Karyotype: diploid<br>Doubling time: 18-38h<br>Grow in “tube-like” structure in low FBS medium	Allen M et al. [2]
SF188	Male, 8-year old	Glioblastoma	Epithelial	Karyotype: hypertriploid<br>Doubling time: 28h	Rutka JT et al. [3]
SF767	Female, 50 year old	Recurrent Glioblastoma		Doubling time: approx. 30h	Maletínská L et al. [4]
U373	Male, 50 year old	Glioblastoma	Glial	Karyotype: dipoid<br>Doubling time: approx. 40h	Bigner DD et al. [5]
U118	Male, 47 -year old	Astrocytoma	Mixed	Karyotype: pentaploid	JAN PONTÉN et al. [6]
SF126	Female, 50 year old	Glioblastoma	Fibroblastic	Karyotype: hypertriploid<br>Doubling time: 29h	Rutka JT et al. [3]
U1242	-	Glioblastoma	Fibroblastic	-	Bongcam-Rudloff E et al. [7]

References

1. Stein GH. (1979). T98G: an anchorage-independent human tumor cell line that exhibits stationary phase G1 arrest in vitro. J Cell Physiol 99, 43-54. 10.1002/jcp.1040990107.
2. Allen M, Bjerke M, Edlund H, Nelander S, Westermark B. Origin of the U87MG glioma cell line: Good news and bad news. (2016). Sci Transl Med 8, 354re3. 10.1126/scitranslmed.aaf6853.
3. Rutka JT, Giblin JR, Dougherty DY, Liu HC, McCulloch JR, Bell CW, Stern RS, Wilson CB, Rosenblum ML. (1987). Establishment and characterization of five cell lines derived from human malignant gliomas. Acta Neuropathol 75, 92-103. 10.1007/BF00686798.
4. Mohapatra G, Kim DH, Feuerstein BG. Detection of multiple gains and losses of genetic material in ten glioma cell lines by comparative genomic hybridization. (1995). Genes Chromosomes Cancer 13, 86-93. 10.1002/gcc.2870130203.
5. Bigner DD, Bigner SH, Pontén J, Westermark B, Mahaley MS, Ruoslahti E, Herschman H, Eng LF, Wikstrand CJ. Heterogeneity of Genotypic and phenotypic characteristics of fifteen permanent cell lines derived from human gliomas. (1981). J Neuropathol Exp Neurol 40, 201-29. 10.1097/00005072-198105000-00001.
6. Pontén J, Macintyre EH. Long term culture of normal and neoplastic human glia. (1968). Acta Pathol Microbiol Scand 74, 465-86. 10.1111/j.1699-0463.1968.tb03502.x.
7. Bongcam-Rudloff E, Nistér M, Betsholtz C, Wang JL, Stenman G, Huebner K, Croce CM, Westermark B. (1991). Human glial fibrillary acidic protein: complementary DNA cloning, chromosome localization, and messenger RNA expression in human glioma cell lines of various phenotypes. Cancer Res 51, 1553-60.
