# Supplementary material for: CD13 expression affects glioma patient survival and influences key functions of human glioblastoma cell lines in vitro
Source: BMC Cancer. 2024 Mar 22;24:369. doi: 10.1186/s12885-024-12113-z (PMC10960415; doi:10.1186/s12885-024-12113-z)
Supplement: Supplementary file 3 — Supplementary Material 3 [file 12885_2024_12113_MOESM3_ESM.pdf]

Supplementary Table 3. Cell seeding numbers

Cell line	8-well chamber (Immunocytochemistry)	96-well plate (MTT assay)	Culture-Insert 24-well plate (Cell scratch assay)
U118	7000 cells/well	6000 cells/well	3.5×10^5 cells/mL
U1242	3000 cells/well	2000 cells/well	2.4×10^5 cells/mL
T98G	4000 cells/well	3000 cells/well	3.5×10^5 cells/mL
SF188	1500 cells/well	1800 cells/well	2.0×10^5 cells/mL
