# Supplementary material for: CD13 expression affects glioma patient survival and influences key functions of human glioblastoma cell lines in vitro
Source: BMC Cancer. 2024 Mar 22;24:369. doi: 10.1186/s12885-024-12113-z (PMC10960415; doi:10.1186/s12885-024-12113-z)
Supplement: Supplementary file 4 — Supplementary Material 4 [file 12885_2024_12113_MOESM4_ESM.pdf]

Supplementary Table 4. CD13 co-expressed genes

gene2	cor	pvalue
TNMD	0.389	1.02E-25
CFH	0.405	6.76E-28
FUCA2	0.32	1.92E-17
TMEM176A	0.337	2.56E-19
TFPI	0.323	8.62E-18
PLXND1	0.44	2.96E-33
SLC22A16	0.35	8.76E-21
PRSS22	0.516	4.90E-47
MEOX1	0.358	1.02E-21
COPZ2	0.342	7.08E-20
ITGA3	0.341	8.80E-20
TNFRSF12A	0.301	1.45E-15
BAIAP2L1	0.525	7.60E-49
ALDH3B1	0.311	1.40E-16
CCL26	0.683	1.26E-93
TBXA2R	0.417	1.05E-29
MYH13	0.361	4.19E-22
TEAD3	0.316	4.44E-17
ST3GAL1	0.419	6.81E-30
UBE3C	0.37	3.52E-23
STAB1	0.322	1.10E-17
HFE	0.349	1.17E-20
FMO1	0.564	1.04E-57
AC080038.1	0.367	6.65E-23
TSPAN9	0.553	3.54E-55
PLAUR	0.545	2.91E-53
DCN	0.562	3.78E-57
PRICKLE3	0.365	1.35E-22
ALOX5	0.326	4.41E-18
DNASE1L1	0.504	1.58E-44
ACPP	0.461	1.07E-36
MARCO	0.407	3.70E-28
SNAI2	0.566	3.78E-58
ADGRA2	0.454	2.11E-35
SERPINB1	0.345	3.01E-20
GLRX2	0.314	8.28E-17
ABCC2	0.356	1.54E-21
EHD2	0.381	1.35E-24
INSRR	0.368	5.37E-23
SH2D2A	0.57	3.75E-59
BAK1	0.347	1.74E-20
GRN	0.4	3.60E-27
CHPF2	0.492	2.91E-42
MAP2K3	0.362	3.32E-22
TMSB10	0.388	1.34E-25
MSR1	0.347	1.86E-20
RAI14	0.311	1.57E-16
AP2S1	0.333	7.56E-19
EPHA3	0.311	1.43E-16

HSPA5	0.418	9.58E-30
H6PD	0.331	1.11E-18
TNFRSF9	0.43	1.10E-31
LTBP1	0.349	1.25E-20
RCN1	0.322	1.12E-17
HEXB	0.429	2.13E-31
NFE2L3	0.341	1.04E-19
LY75	0.304	7.39E-16
DCBLD2	0.421	3.16E-30
SOAT1	0.317	3.83E-17
PRDM1	0.323	9.30E-18
SEC61A1	0.323	9.39E-18
SLC2A3	0.308	3.46E-16
GNA15	0.361	3.81E-22
LZTS1	0.339	1.54E-19
CASP8	0.35	8.45E-21
CTSA	0.344	4.22E-20
CNN2	0.474	7.36E-39
SBNO2	0.338	2.02E-19
PDIA5	0.49	6.78E-42
TRAM1	0.348	1.47E-20
TNFRSF1A	0.329	1.99E-18
HYAL2	0.315	5.67E-17
LAPTM4A	0.341	8.26E-20
BCL3	0.522	3.55E-48
GAL	0.304	8.30E-16
ATP1B3	0.318	3.32E-17
RAB27A	0.537	1.44E-51
WIPI1	0.484	9.60E-41
P4HA2	0.467	1.03E-37
FCGR2B	0.366	1.04E-22
NOTCH3	0.321	1.44E-17
TEAD2	0.319	2.37E-17
CA12	0.346	2.49E-20
LMAN1	0.348	1.37E-20
MYDGF	0.383	6.97E-25
SEMA3A	0.43	1.26E-31
FNDC3B	0.492	3.25E-42
ACTB	0.333	7.58E-19
MOCOS	0.395	1.50E-26
REXO2	0.353	3.67E-21
ICAM3	0.358	1.06E-21
IL4R	0.383	6.41E-25
CAPN6	0.442	1.71E-33
SLC25A43	0.31	1.79E-16
FBLN1	0.392	3.58E-26
CST7	0.365	1.29E-22
FAP	0.758	2.64E-126
P2RY10	0.395	1.79E-26
SDF4	0.342	6.38E-20
FKBP7	0.427	3.89E-31

PUM3	0.312	1.17E-16
ATP8B1	0.314	6.83E-17
MPP4	0.339	1.58E-19
COL19A1	0.378	3.41E-24
PLOD1	0.548	7.95E-54
FCN1	0.333	8.20E-19
SLC25A24	0.362	3.14E-22
B4GALT1	0.526	4.89E-49
TMED2	0.331	1.33E-18
FTL	0.326	4.22E-18
ADAMTS2	0.458	3.30E-36
MMP2	0.307	3.83E-16
GNAO1	-0.302	1.20E-15
NID2	0.364	1.61E-22
TFAP2C	0.404	9.80E-28
EDEM2	0.311	1.52E-16
FKBP1A	0.374	9.59E-24
SIRPG	0.388	1.56E-25
FXYD5	0.381	1.37E-24
GANAB	0.326	4.62E-18
ICAM1	0.357	1.17E-21
LYZ	0.396	1.02E-26
EFNB1	0.331	1.08E-18
DTX2	0.339	1.70E-19
LAMB1	0.611	4.75E-70
CMTM6	0.328	2.77E-18
NANS	0.302	1.21E-15
IL11	0.537	1.97E-51
ITPR3	0.366	9.16E-23
NRP1	0.503	2.45E-44
KCNK6	0.343	5.64E-20
HSD3B7	0.31	1.89E-16
MAGEB2	0.355	2.28E-21
MMP11	0.347	1.80E-20
SUSD2	0.373	1.25E-23
LGALS1	0.521	5.43E-48
KDELR3	0.666	2.15E-87
LMF2	0.343	5.68E-20
HMOX1	0.311	1.56E-16
TSPO	0.339	1.55E-19
MYH9	0.367	7.37E-23
NCF4	0.311	1.43E-16
IL2RB	0.385	3.17E-25
GZMH	0.349	1.10E-20
PYGL	0.313	9.76E-17
PLEK2	0.336	3.41E-19
LGMN	0.302	1.34E-15
BDKRB1	0.497	3.91E-43
PLTP	0.373	1.40E-23
MMP9	0.333	7.64E-19
PROCR	0.421	3.33E-30

CD40	0.391	5.44E-26
R3HDML	0.397	8.15E-27
SLA2	0.354	2.89E-21
TPD52L2	0.31	2.16E-16
CTSZ	0.508	1.99E-45
NTSR1	0.384	5.14E-25
SLC17A9	0.512	2.83E-46
MYL12A	0.432	6.64E-32
MXRA5	0.608	4.20E-69
TLR8	0.327	3.63E-18
AMMECR1	0.369	4.23E-23
SRPX	0.335	4.08E-19
PLP2	0.35	8.48E-21
ELF4	0.458	4.08E-36
ASB9	0.375	7.70E-24
SLC35A2	0.376	5.53E-24
SMS	0.319	2.09E-17
TIMP1	0.426	5.55E-31
SRPX2	0.495	9.24E-43
GLA	0.385	4.18E-25
KLF5	0.332	8.99E-19
ACOD1	0.329	2.24E-18
TRADD	0.315	6.18E-17
AQP9	0.365	1.43E-22
CD276	0.507	3.09E-45
FAH	0.341	9.95E-20
EHD4	0.377	4.19E-24
PDGFRL	0.436	1.35E-32
CPQ	0.339	1.47E-19
SFRP1	0.304	7.34E-16
SH2D4A	0.35	7.75E-21
TNFRSF10A	0.498	2.22E-43
KCTD9	0.393	2.62E-26
MAN2B1	0.37	3.42E-23
KCNN4	0.364	1.93E-22
NUCB1	0.327	3.23E-18
RELB	0.355	2.21E-21
FCGRT	0.33	1.38E-18
RETN	0.408	2.68E-28
SYDE1	0.329	1.77E-18
SLC1A5	0.398	5.50E-27
AC011462.1	0.392	4.29E-26
CEACAM4	0.357	1.21E-21
NKG7	0.349	1.24E-20
CD33	0.308	3.32E-16
KDELR1	0.335	4.43E-19
CLEC11A	0.524	9.86E-49
TFPI2	0.362	2.88E-22
TWISTNB	0.322	1.14E-17
CAV2	0.326	4.38E-18
CAV1	0.436	1.31E-32

CPED1	0.577	6.41E-61
GRB10	0.375	6.33E-24
NPTX2	0.312	1.23E-16
PCOLCE	0.738	2.31E-116
IMPDH1	0.553	4.39E-55
SERPINE1	0.608	3.95E-69
PLOD3	0.612	2.40E-70
MEOX2	0.326	4.07E-18
RARRES2	0.382	9.53E-25
AHR	0.329	1.84E-18
TMEM176B	0.313	1.05E-16
TBL2	0.426	4.94E-31
LIMK1	0.384	4.74E-25
ENG	0.465	2.84E-37
PDLIM1	0.34	1.19E-19
CXCL12	0.31	1.84E-16
STN1	0.347	1.83E-20
MAP3K8	0.33	1.73E-18
PFN1	0.374	1.02E-23
CPD	0.385	3.45E-25
CCL7	0.317	3.40E-17
COL1A1	0.643	1.08E-79
LRRC59	0.338	2.08E-19
FAM20A	0.605	2.91E-68
RAB34	0.31	2.19E-16
SULT1E1	0.494	1.37E-42
PF4V1	0.352	4.42E-21
BST1	0.367	6.68E-23
CTSC	0.462	7.03E-37
MS4A6A	0.341	1.03E-19
MS4A4A	0.381	1.38E-24
TMEM109	0.355	2.48E-21
MDK	0.381	1.18E-24
SLC22A18	0.356	1.60E-21
TCIRG1	0.378	2.69E-24
P3H3	0.309	2.27E-16
CLEC2B	0.458	3.95E-36
ELK3	0.394	2.32E-26
SH2B3	0.425	7.97E-31
LTBR	0.361	4.39E-22
MGP	0.323	7.83E-18
ARHGDIB	0.333	6.72E-19
VDR	0.596	7.33E-66
COL12A1	0.304	7.90E-16
DSE	0.371	2.25E-23
MAN1A1	0.409	1.58E-28
TREML2	0.345	3.66E-20
CRYBG1	0.307	3.65E-16
VNN1	0.315	6.28E-17
VNN2	0.314	6.95E-17
TRIM38	0.328	2.55E-18

SMOC2	0.433	4.48E-32
LAMA4	0.401	2.30E-27
C7	0.443	1.16E-33
DAP	0.304	8.50E-16
LOX	0.394	2.31E-26
GZMK	0.35	8.06E-21
ST8SIA4	0.355	2.45E-21
RARS	0.32	1.82E-17
PDGFRB	0.537	1.44E-51
CDX1	0.38	1.52E-24
SSR3	0.317	3.43E-17
MOB1A	0.31	2.14E-16
CCL20	0.326	4.19E-18
ACTR3	0.356	1.78E-21
STEAP3	0.353	3.85E-21
CYTIP	0.321	1.44E-17
ACVR1	0.387	2.15E-25
NRBP1	0.342	7.72E-20
ITGA4	0.486	3.28E-41
LOXL3	0.411	7.70E-29
DOK1	0.319	2.29E-17
EVA1A	0.472	1.21E-38
FN1	0.537	1.96E-51
IGFBP5	0.339	1.49E-19
GGCX	0.331	1.36E-18
IL1R2	0.316	4.28E-17
IL1R1	0.332	8.86E-19
FHL2	0.365	1.32E-22
MLPH	0.7	4.69E-100
HDLBP	0.316	5.34E-17
SDC1	0.62	1.77E-72
KYNU	0.389	9.20E-26
WIPF1	0.343	5.29E-20
ICMT	0.321	1.29E-17
ERRFI1	0.39	8.32E-26
CAPZA1	0.346	2.64E-20
PRG4	0.495	8.20E-43
NCF2	0.341	9.46E-20
OLFML3	0.398	7.05E-27
CD2	0.386	3.02E-25
NID1	0.517	3.42E-47
LGALS8	0.307	3.85E-16
MFAP2	0.361	4.00E-22
UAP1	0.36	5.69E-22
ECE1	0.341	9.91E-20
P3H1	0.628	5.25E-75
ESYT2	0.308	3.44E-16
CTSD	0.335	4.82E-19
MMP8	0.309	2.72E-16
VAMP8	0.313	9.23E-17
RPN2	0.337	2.76E-19

SPP1	0.328	2.70E-18
MFSD1	0.351	6.17E-21
PTPA	0.315	6.51E-17
SLC46A2	0.306	4.51E-16
GALNT12	0.319	2.07E-17
ALG2	0.379	2.28E-24
NPC2	0.377	3.96E-24
LTBP2	0.369	4.34E-23
TGFB3	0.319	2.16E-17
TMEM214	0.483	1.61E-40
TNFSF11	0.302	1.17E-15
TGFBI	0.637	8.92E-78
GLT8D2	0.559	1.54E-56
DUSP4	0.338	1.82E-19
TNFRSF10B	0.368	5.87E-23
SCPEP1	0.364	1.73E-22
TSHZ3	0.355	2.47E-21
PLBD1	0.513	2.48E-46
CSTA	0.383	6.79E-25
CCR2	0.469	5.48E-38
XPNPEP2	0.483	1.20E-40
PAEP	0.352	5.14E-21
FKBP9	0.572	1.05E-59
TWIST1	0.521	4.01E-48
CALD1	0.383	6.07E-25
PLAU	0.381	1.20E-24
SRGN	0.314	6.88E-17
BICC1	0.366	9.32E-23
P4HA1	0.381	1.26E-24
NME2P1	0.317	3.64E-17
PRDX4	0.324	6.46E-18
MMP19	0.392	3.58E-26
COL10A1	0.638	3.53E-78
SERPINA7	0.554	2.16E-55
TNFAIP6	0.352	4.54E-21
RAB38	0.411	9.77E-29
CHPF	0.396	1.31E-26
PIGT	0.385	4.04E-25
SNAI1	0.528	1.67E-49
PMEPA1	0.44	3.80E-33
F13A1	0.395	1.47E-26
TREM1	0.333	6.97E-19
RUNX2	0.301	1.71E-15
EREG	0.343	5.74E-20
EFNB2	0.362	3.19E-22
CD70	0.37	3.32E-23
VASP	0.385	3.15E-25
CD93	0.38	1.51E-24
BFSP1	0.33	1.74E-18
S1PR4	0.401	2.38E-27
CCR7	0.347	1.75E-20

RRAS	0.387	1.79E-25
ASL	0.307	4.25E-16
WNK4	0.36	4.94E-22
SLC10A3	0.518	1.93E-47
FGFRL1	0.318	2.96E-17
ADGRE2	0.398	5.48E-27
GNG11	0.367	7.19E-23
PTPN12	0.384	4.74E-25
STEAP4	0.402	1.63E-27
SDF2L1	0.314	7.64E-17
A4GALT	0.395	1.59E-26
RAC2	0.513	1.89E-46
LIF	0.301	1.51E-15
APOBEC3F	0.329	1.78E-18
CALU	0.532	2.57E-50
OPN1SW	0.339	1.45E-19
MYO1B	0.45	8.86E-35
CLN6	0.306	5.17E-16
ISLR	0.423	1.75E-30
LOXL1	0.488	1.85E-41
CD68	0.377	4.56E-24
SIGLEC9	0.317	3.46E-17
RNASE1	0.329	1.76E-18
CRACR2A	0.444	8.18E-34
SAT1	0.328	2.66E-18
NECTIN2	0.394	1.99E-26
ATP8B3	0.306	5.31E-16
PLVAP	0.341	9.54E-20
COLGALT1	0.569	5.54E-59
ARPC1B	0.578	4.62E-61
PXDN	0.437	9.93E-33
GDF15	0.302	1.13E-15
LSP1	0.49	6.57E-42
TNNT3	0.483	1.68E-40
COL5A1	0.708	2.32E-103
EPS8L1	0.329	1.89E-18
LILRB2	0.393	2.73E-26
MGAT1	0.386	2.36E-25
ANO1	0.583	1.58E-62
IL13RA1	0.319	2.32E-17
TNS4	0.329	1.90E-18
CHSY1	0.405	6.67E-28
MATN3	0.671	5.26E-89
CLEC10A	0.423	1.68E-30
TESMIN	0.321	1.49E-17
ALOX5AP	0.337	2.89E-19
POSTN	0.355	1.95E-21
C1QTNF6	0.534	7.75E-51
GIMAP6	0.312	1.28E-16
LYVE1	0.324	7.17E-18
LOXL2	0.642	1.97E-79

EDEM1	0.402	1.70E-27
PTPN22	0.369	3.86E-23
FKBP11	0.488	1.41E-41
LDHA	0.363	2.19E-22
IL2RA	0.423	1.30E-30
SLC43A3	0.365	1.15E-22
COL4A2	0.367	7.15E-23
KDELC1	0.314	8.41E-17
STT3A	0.368	5.34E-23
OSTF1	0.323	8.68E-18
CTSL	0.4	3.38E-27
CEMIP2	0.469	4.47E-38
P2RX4	0.321	1.58E-17
TES	0.413	4.68E-29
MDFIC	0.339	1.43E-19
CD63	0.319	2.44E-17
TEC	0.451	4.84E-35
FHOD1	0.46	1.92E-36
FAM129A	0.418	8.50E-30
LAMC1	0.575	2.06E-60
CKAP4	0.661	1.70E-85
LCP1	0.345	3.08E-20
KDELR2	0.518	1.75E-47
BZW2	0.312	1.33E-16
MYO1G	0.407	3.17E-28
ADAMTS7	0.323	8.23E-18
GALNT5	0.371	2.30E-23
IL10	0.353	3.50E-21
TXN	0.343	6.23E-20
FAM129B	0.427	3.17E-31
SLC31A1	0.308	3.16E-16
SIT1	0.366	1.02E-22
CD72	0.313	8.91E-17
LRRC32	0.337	2.41E-19
PI15	0.339	1.54E-19
SDCBP	0.312	1.19E-16
POU2F3	0.354	2.51E-21
THBS1	0.309	2.48E-16
ITGA11	0.358	9.95E-22
STRA6	0.333	7.65E-19
CYP1B1	0.402	2.00E-27
RAB1A	0.322	1.20E-17
EMILIN1	0.674	3.60E-90
MYOF	0.314	6.85E-17
CALHM2	0.435	2.00E-32
GLCE	0.313	8.85E-17
CILP	0.476	3.19E-39
FGF5	0.377	4.28E-24
GPAT3	0.317	3.95E-17
NAAA	0.363	2.18E-22
CASP6	0.306	4.62E-16

LEF1	0.347	1.73E-20
SLC39A8	0.39	8.04E-26
FBN2	0.322	1.22E-17
C1RL	0.311	1.68E-16
CD27	0.327	3.32E-18
GLIPR1	0.35	7.42E-21
LUM	0.626	2.58E-74
KERA	0.491	4.69E-42
DHH	0.384	5.66E-25
ACVRL1	0.444	8.93E-34
GPR84	0.311	1.62E-16
ESYT1	0.307	3.88E-16
BCL2A1	0.314	7.90E-17
TLNRD1	0.317	3.60E-17
ADAMTS17	0.308	3.39E-16
MCTP2	0.458	3.63E-36
FURIN	0.505	1.06E-44
IQGAP1	0.328	2.72E-18
PMM2	0.427	4.39E-31
CMTM3	0.303	1.02E-15
CDH11	0.372	1.60E-23
GALNS	0.357	1.29E-21
IMPA2	0.307	4.30E-16
SLC16A3	0.465	2.41E-37
SECTM1	0.311	1.39E-16
TNFRSF11A	0.315	5.98E-17
PMAIP1	0.399	4.41E-27
P3H4	0.351	5.93E-21
IGFBP4	0.517	3.83E-47
FKBP10	0.379	2.48E-24
COL6A1	0.761	3.62E-128
COL6A2	0.75	2.64E-122
IL19	0.352	5.29E-21
NLRP12	0.342	7.33E-20
RCN3	0.718	1.88E-107
EPHA2	0.395	1.61E-26
ARHGEF19	0.388	1.32E-25
SH3BGRL3	0.414	3.25E-29
EVA1B	0.516	4.86E-47
FCN3	0.411	9.21E-29
HSPG2	0.384	5.17E-25
ITGA10	0.334	5.03E-19
FCGR2A	0.409	2.05E-28
CRABP2	0.358	1.05E-21
ECM1	0.529	9.73E-50
CTSK	0.532	1.91E-50
CERS2	0.312	1.26E-16
S100A8	0.354	2.74E-21
TPM3	0.466	1.90E-37
SLC39A1	0.336	3.08E-19
GALNT2	0.322	1.17E-17

ARF1	0.322	1.00E-17
CNIH3	0.325	5.72E-18
PTPN7	0.302	1.36E-15
PDIA6	0.387	2.08E-25
TRIM43B	0.478	1.08E-39
TRIM43	0.465	2.46E-37
MALL	0.624	7.02E-74
RALB	0.406	4.55E-28
SLC20A1	0.477	1.81E-39
AL078621.1	0.308	3.11E-16
SUMF1	0.34	1.23E-19
OSBPL10	0.315	5.67E-17
SHQ1	0.353	4.30E-21
NFKBIZ	0.306	4.63E-16
COL8A1	0.305	6.92E-16
MANF	0.388	1.59E-25
OSMR	0.391	5.67E-26
GZMA	0.362	3.31E-22
TSLP	0.315	6.37E-17
TNFAIP8	0.308	3.03E-16
YIPF5	0.329	1.95E-18
DOK3	0.308	3.20E-16
PPP1R18	0.323	7.96E-18
TPBG	0.468	8.00E-38
ARHGAP18	0.349	9.70E-21
ABRACL	0.34	1.31E-19
LINC00525	0.368	6.30E-23
SYTL5	0.416	1.77E-29
GPR174	0.484	8.75E-41
IL2RG	0.339	1.42E-19
NXF3	0.304	8.28E-16
ZNF185	0.376	5.51E-24
DOK2	0.469	5.07E-38
GINS4	0.336	3.13E-19
VLDLR	0.322	1.02E-17
PLIN2	0.386	2.94E-25
SURF4	0.501	5.98E-44
GBGT1	0.337	2.47E-19
PTGES	0.692	7.78E-97
ITPRIP	0.317	3.53E-17
ADAM12	0.495	8.63E-43
SERPINH1	0.731	3.41E-113
P4HA3	0.859	8.89E-197
ST14	0.348	1.52E-20
MPZL2	0.355	2.22E-21
FERMT3	0.319	2.17E-17
CLEC1A	0.378	3.16E-24
ITGB1	0.499	1.32E-43
LATS2	0.327	2.99E-18
VEGFC	0.392	3.62E-26
PRSS23	0.378	3.16E-24

SEC24D	0.556	1.09E-55
PLBD2	0.314	8.56E-17
EXT2	0.337	2.83E-19
ADAMTS12	0.472	1.62E-38
ADAM8	0.353	3.58E-21
TDO2	0.441	2.06E-33
PABPC3	0.384	5.50E-25
PSTPIP2	0.319	2.47E-17
PELO	0.354	2.90E-21
PLOD2	0.337	2.40E-19
DAB2	0.309	2.20E-16
CAST	0.352	5.19E-21
TMEM87B	0.394	2.05E-26
PLA2R1	0.433	4.94E-32
RBMS1	0.425	6.60E-31
CD96	0.354	2.53E-21
CMTM7	0.383	6.54E-25
CD8A	0.327	3.42E-18
HS3ST3A1	0.355	1.95E-21
UBASH3B	0.499	1.37E-43
CERS3	0.46	1.62E-36
ABCA9	0.473	8.35E-39
ABCA6	0.307	3.50E-16
ABCA10	0.311	1.74E-16
CNTNAP3B	0.391	5.06E-26
LY96	0.359	6.95E-22
DKK2	0.423	1.27E-30
SLC7A7	0.338	1.91E-19
VSIG4	0.367	7.86E-23
PDIA4	0.485	5.91E-41
CLIC2	0.352	4.80E-21
BATF	0.361	4.70E-22
ZDHHC5	0.376	5.99E-24
FRRS1	0.423	1.40E-30
FAM92A1P1	0.303	1.07E-15
NTAN1	0.438	6.55E-33
MMP14	0.584	9.55E-63
FZD1	0.57	3.33E-59
KCNJ15	0.359	7.69E-22
TNFRSF14	0.304	7.36E-16
RER1	0.301	1.61E-15
CD1D	0.454	2.09E-35
CD1C	0.319	2.16E-17
TAGLN2	0.356	1.79E-21
SLAMF8	0.454	1.56E-35
DUSP23	0.424	1.07E-30
FCER1G	0.41	1.11E-28
IFNGR2	0.393	2.74E-26
STC1	0.604	4.70E-68
PRAC1	0.481	3.77E-40
HOXB13	0.324	7.42E-18

C1QC	0.319	2.56E-17
RUNX1	0.406	5.34E-28
CLDN14	0.306	5.17E-16
ADPGK	0.406	5.08E-28
C1R	0.369	4.51E-23
MED8	0.316	4.49E-17
SPON2	0.659	8.11E-85
ZYX	0.308	3.44E-16
PTGIR	0.335	4.02E-19
G6PD	0.336	3.75E-19
ITGB2	0.303	1.08E-15
MPZL3	0.321	1.54E-17
JAML	0.307	4.36E-16
CD3G	0.342	6.84E-20
CXCR5	0.316	4.84E-17
SHC1	0.559	1.82E-56
LMNA	0.483	1.64E-40
HK3	0.415	2.54E-29
LY6E	0.31	1.78E-16
MGAT4B	0.313	9.08E-17
PSMC2	0.336	3.48E-19
PLXDC1	0.35	8.39E-21
CYGB	0.468	6.00E-38
ITGA5	0.621	8.74E-73
ASGR2	0.361	4.49E-22
SELENON	0.367	7.06E-23
FBLIM1	0.664	9.99E-87
LAPTM5	0.301	1.63E-15
MXRA8	0.366	1.02E-22
DIRAS3	0.325	5.30E-18
SLC30A7	0.316	4.62E-17
SLAMF6	0.373	1.24E-23
OLFML2B	0.573	5.12E-60
DENND2D	0.307	3.60E-16
SNED1	0.564	1.24E-57
TFB2M	0.303	1.04E-15
IL24	0.321	1.30E-17
PQLC3	0.493	2.30E-42
CTSS	0.363	2.52E-22
RNF149	0.39	7.91E-26
S100A11	0.504	1.55E-44
S100A9	0.39	8.47E-26
ANTXR2	0.505	8.14E-45
COL6A3	0.661	1.43E-85
EOGT	0.324	6.88E-18
FSTL1	0.381	1.16E-24
ARPC2	0.405	6.71E-28
TGFBR2	0.361	3.86E-22
TRAT1	0.302	1.10E-15
PYHIN1	0.37	2.86E-23
AIM2	0.447	2.72E-34

CTLA4	0.496	4.26E-43
ICOS	0.34	1.35E-19
CD200R1	0.353	3.44E-21
CDCP1	0.449	1.15E-34
RPN1	0.323	9.25E-18
SPRY1	0.304	8.31E-16
PPM1M	0.314	7.58E-17
F2RL2	0.338	2.19E-19
GPX8	0.563	1.93E-57
ADGRF2	0.31	2.07E-16
CALHM4	0.322	1.08E-17
STEAP1	0.484	1.08E-40
COL1A2	0.667	8.22E-88
BRI3	0.325	5.95E-18
CTSB	0.515	7.72E-47
CTHRC1	0.613	1.86E-70
DCSTAMP	0.457	5.13E-36
HGSNAT	0.403	1.42E-27
FBP1	0.352	4.41E-21
IKBIP	0.505	1.11E-44
FBN1	0.429	1.77E-31
CLMP	0.535	5.17E-51
C2	0.334	5.46E-19
MFAP4	0.305	6.76E-16
CLEC4D	0.346	2.38E-20
TMED3	0.361	4.29E-22
HSP90B1	0.371	2.20E-23
PPIB	0.405	6.19E-28
ANPEP	1	0
STAT6	0.308	3.15E-16
C15orf48	0.346	2.82E-20
CNPY4	0.382	9.43E-25
PDIA3	0.424	1.01E-30
TMEM92	0.34	1.20E-19
PRRX2	0.538	1.38E-51
CD3D	0.386	2.82E-25
TPM4	0.467	1.00E-37
TUBA1C	0.393	3.09E-26
SERTAD3	0.311	1.53E-16
LAIR1	0.331	1.17E-18
CDC42EP5	0.607	6.36E-69
IGFBP6	0.454	1.55E-35
SOAT2	0.464	3.64E-37
CD300C	0.313	1.07E-16
SCARA5	0.418	8.76E-30
VASN	0.304	7.88E-16
ARF4	0.305	5.95E-16
BDKRB2	0.338	1.99E-19
MLKL	0.363	2.21E-22
RHOH	0.305	5.89E-16
REEP4	0.359	8.22E-22

BMP1	0.527	3.19E-49
SERINC2	0.301	1.45E-15
COL3A1	0.607	6.78E-69
IL7R	0.438	7.18E-33
PPIC	0.388	1.43E-25
SIGLEC7	0.331	1.20E-18
SLC49A3	0.323	9.17E-18
CHST14	0.351	6.23E-21
APEX2	0.315	6.63E-17
LMAN2	0.333	6.72E-19
SLC50A1	0.394	2.35E-26
RNASE2	0.326	4.16E-18
COL22A1	0.324	7.74E-18
CD52	0.361	4.04E-22
ZNF280A	0.398	6.36E-27
AGPAT2	0.37	3.56E-23
LIMS1	0.372	1.70E-23
GUSB	0.395	1.78E-26
GLB1	0.419	6.59E-30
B3GNT2	0.43	1.47E-31
OR2A20P	0.407	3.58E-28
CD14	0.36	5.68E-22
SMAGP	0.547	1.13E-53
EMB	0.37	3.10E-23
HOXB9	0.344	3.77E-20
HTRA3	0.438	8.16E-33
OSCAR	0.303	9.37E-16
CAVIN3	0.732	6.12E-114
HAS2	0.315	6.02E-17
LRRC8E	0.38	1.74E-24
FPR2	0.337	2.73E-19
C11orf24	0.369	4.00E-23
SIGLEC17P	0.48	5.71E-40
C1GALT1C1	0.32	1.96E-17
TMEM37	0.319	2.32E-17
LRG1	0.415	2.25E-29
NAT1	0.327	3.02E-18
MROH2B	0.515	9.43E-47
PTGER4	0.464	3.32E-37
LRRC15	0.358	9.77E-22
CD8B	0.402	1.66E-27
CXCR6	0.402	1.98E-27
CEBPB	0.378	2.83E-24
STARD5	0.313	8.74E-17
SMPDL3A	0.328	2.74E-18
EFEMP2	0.363	2.09E-22
RPL12P13	0.355	1.90E-21
RAB43	0.341	8.32E-20
RARG	0.328	2.69E-18
MYEOV	0.652	1.04E-82
MYD88	0.338	2.16E-19

MIR4435-2H	0.321	1.55E-17
GXYLT2	0.311	1.59E-16
EVC2	0.319	2.10E-17
BNC2	0.357	1.20E-21
PPP1R3B	0.34	1.26E-19
C1QB	0.322	1.24E-17
C1QA	0.339	1.69E-19
EHBP1L1	0.31	2.15E-16
TNFRSF10D	0.639	1.85E-78
TNFRSF10C	0.308	2.89E-16
CD7	0.304	8.65E-16
PLK3	0.31	2.13E-16
DIRC1	0.507	2.86E-45
SLC16A13	0.396	1.24E-26
RPL35P9	0.323	8.66E-18
CMKLR1	0.342	7.06E-20
FAM241A	0.364	1.75E-22
CD248	0.586	4.32E-63
GPR171	0.304	8.39E-16
CATSPER1	0.344	4.57E-20
LRRC25	0.361	3.81E-22
CLCF1	0.312	1.31E-16
KCNE3	0.321	1.53E-17
FOSL1	0.424	9.36E-31
TMEM70	0.368	5.14E-23
TUBB6	0.33	1.66E-18
TMEM39A	0.355	2.03E-21
OR10AC1	0.326	3.82E-18
B3GNT5	0.341	1.03E-19
FOXL1	0.354	2.54E-21
METRNL	0.375	8.36E-24
KRT18P28	0.33	1.38E-18
TCIM	0.322	1.19E-17
PHETA2	0.504	1.71E-44
B3GNT8	0.519	1.53E-47
HIC1	0.462	8.34E-37
CAVIN1	0.445	4.99E-34
CD163	0.379	2.48E-24
CD151	0.35	7.77E-21
FAM20C	0.438	6.21E-33
BET1L	0.354	2.92E-21
CALHM5	0.313	1.07E-16
KDELC2	0.394	2.50E-26
PRSS36	0.323	9.57E-18
RPL10P16	0.348	1.62E-20
CD28	0.337	2.96E-19
ARMC10P1	0.302	1.21E-15
THBD	0.357	1.27E-21
MSC	0.334	6.35E-19
SPTY2D1	0.327	3.50E-18
FUCA1	0.345	3.13E-20

CALR	0.381	1.08E-24
APOBEC3B	0.398	6.83E-27
FOXS1	0.462	7.77E-37
MYADM	0.419	6.12E-30
ABCA13	0.308	2.86E-16
B3GNT3	0.456	7.42E-36
GPBAR1	0.427	4.04E-31
CCR8	0.346	2.53E-20
SSC5D	0.346	2.53E-20
TMEM150B	0.406	4.73E-28
FO393411.1	0.301	1.45E-15
TPT1P10	0.305	6.80E-16
FZD2	0.398	6.43E-27
PRF1	0.326	3.81E-18
OR51E1	0.322	1.07E-17
PDIA3P1	0.341	8.49E-20
FCRL6	0.46	1.45E-36
CCL13	0.461	1.11E-36
TMEM45A	0.347	1.91E-20
RAP2B	0.385	3.94E-25
RNF135	0.341	1.01E-19
TNFSF15	0.308	2.84E-16
PHLDA2	0.545	2.94E-53
COPG1	0.315	6.13E-17
SLC35C1	0.496	5.52E-43
OR4C6	0.717	6.61E-107
CREB3L2	0.386	2.48E-25
BACE2	0.451	4.76E-35
C1S	0.353	3.45E-21
BGN	0.507	3.89E-45
FES	0.372	1.85E-23
TSKU	0.459	2.92E-36
ANXA2	0.467	1.13E-37
FAM230E	0.514	1.19E-46
LCK	0.386	2.46E-25
COL18A1	0.477	1.49E-39
SRPRA	0.453	2.23E-35
MCEMP1	0.391	5.70E-26
TPM3P8	0.373	1.39E-23
UPP1	0.387	2.13E-25
CCR4	0.382	7.82E-25
KIRREL1	0.494	1.37E-42
ARSI	0.405	5.83E-28
SMTN	0.304	8.33E-16
AC026410.1	0.336	2.99E-19
OAF	0.566	3.85E-58
ALDH1A3	0.399	4.37E-27
PKP3	0.414	3.79E-29
TMEM255B	0.332	9.42E-19
SOCS3	0.344	4.11E-20
TMEM173	0.318	2.65E-17

RPL7P26	0.317	3.63E-17
TMED9	0.35	7.72E-21
FAM43A	0.301	1.54E-15
IFITM2	0.327	3.56E-18
TCEAL9	0.337	2.82E-19
TCN2	0.329	2.24E-18
ACTBP7	0.361	3.67E-22
P4HB	0.495	8.56E-43
SLC52A2	0.308	3.08E-16
RPL12P4	0.312	1.14E-16
AIDA	0.309	2.40E-16
AC012085.1	0.303	1.05E-15
EDARADD	0.522	2.93E-48
CD300E	0.394	2.39E-26
TPI1P3	0.32	1.77E-17
CXCR3	0.387	1.80E-25
TNFRSF4	0.424	1.09E-30
TNFRSF18	0.424	9.33E-31
LILRA5	0.342	7.09E-20
GCNT1	0.343	6.12E-20
FPR3	0.353	3.41E-21
COL4A1	0.354	3.22E-21
RPL7P48	0.315	5.85E-17
GJA4	0.322	1.03E-17
POTEG	0.359	6.53E-22
AGMO	0.324	6.95E-18
SBK2	0.5	6.85E-44
TMSB4XP8	0.313	1.06E-16
TRPV2	0.409	1.67E-28
CXXC1P1	0.307	4.17E-16
KCTD9P3	0.365	1.51E-22
RAB42	0.348	1.34E-20
POTEE	0.353	3.98E-21
ACTBP11	0.359	7.74E-22
FAM83G	0.39	7.76E-26
DPYD	0.374	8.74E-24
PARVB	0.329	1.94E-18
TMSB4XP2	0.303	1.09E-15
RPL10AP2	0.308	2.95E-16
GJB3	0.417	1.29E-29
PCDH18	0.398	5.67E-27
S100A4	0.687	5.92E-95
OR10J3	0.35	8.23E-21
PTPN1	0.307	3.59E-16
EPHB4	0.418	8.84E-30
S100A5	0.311	1.74E-16
RFX8	0.611	4.19E-70
MME	0.43	1.31E-31
LAMA2	0.426	5.63E-31
POTEF	0.368	6.44E-23
MMP1	0.542	1.32E-52

POTEI	0.362	3.21E-22
PDLIM7	0.333	7.58E-19
TMEM26	0.459	2.94E-36
CASP4	0.426	5.89E-31
SIGLEC15	0.693	2.27E-97
SND1	0.306	5.39E-16
SERPINA1	0.315	6.46E-17
KANK2	0.347	1.84E-20
GTF2E2	0.381	1.28E-24
SVIL	0.355	1.89E-21
C5AR1	0.357	1.16E-21
GSTK1	0.304	6.97E-16
SLC2A10	0.33	1.61E-18
DPP4	0.439	5.81E-33
FAM114A1	0.479	7.27E-40
PTMAP2	0.329	2.09E-18
CFD	0.305	6.92E-16
MYO1C	0.506	5.28E-45
CHSY3	0.434	2.75E-32
TOR4A	0.4	3.75E-27
PTMAP9	0.326	4.00E-18
FKBP1C	0.359	6.43E-22
TMEM239	0.343	5.11E-20
PIM3	0.313	8.79E-17
TPM2	0.335	4.60E-19
ITGBL1	0.33	1.39E-18
PAPSS2	0.35	7.84E-21
GLMP	0.425	7.13E-31
APCDD1L	0.477	2.04E-39
CD247	0.316	5.08E-17
CD3E	0.369	4.83E-23
OSTC	0.411	8.93E-29
NAGA	0.367	7.35E-23
TGM2	0.326	4.09E-18
AC004988.1	0.573	7.12E-60
CR1	0.312	1.10E-16
FCGR3A	0.33	1.60E-18
TMEM244	0.557	6.08E-56
HSP90B3P	0.329	1.95E-18
ARMCX7P	0.317	4.15E-17
COL5A2	0.607	9.02E-69
COL15A1	0.594	2.05E-65
LAYN	0.404	8.36E-28
POTEKP	0.324	6.30E-18
LILRB3	0.351	6.31E-21
ARHGEF34P	0.4	3.47E-27
CFI	0.357	1.17E-21
AC121338.1	0.511	6.40E-46
ITPRIPL2	0.355	2.14E-21
MIR635	0.353	4.11E-21
MIR216A	0.349	1.17E-20

IGLV3-27	0.453	2.29E-35
TRBV7-3	0.301	1.55E-15
TRBV5-1	0.301	1.66E-15
TRBV19	0.438	6.10E-33
TRBV20-1	0.367	7.28E-23
TRBV28	0.372	1.84E-23
TRAV6	0.358	9.45E-22
TRAV10	0.419	6.16E-30
TRAV12-2	0.339	1.57E-19
TRAV8-4	0.335	3.91E-19
TRAV14DV4	0.387	2.08E-25
TRAV9-2	0.323	8.60E-18
TRAV26-1	0.336	3.23E-19
TRAJ23	0.588	8.16E-64
TRAJ22	0.479	8.71E-40
IGHG2	0.328	2.80E-18
IGHV1-46	0.441	2.58E-33
IGHV3-49	0.325	5.02E-18
RPL31P11	0.311	1.44E-16
AC069218.1	0.381	1.30E-24
ARHGEF35	0.542	1.22E-52
YWHAZP2	0.334	5.50E-19
IFITM9P	0.329	2.24E-18
AC005521.1	0.313	9.77E-17
AC108073.1	0.318	3.06E-17
FTH1P12	0.324	6.33E-18
AC022210.1	0.304	8.68E-16
ANXA2P1	0.366	1.02E-22
GAPDHP28	0.316	5.22E-17
FTH1P3	0.311	1.51E-16
ACTN4P1	0.35	9.00E-21
PPIAP16	0.314	8.20E-17
RPLP0P6	0.308	2.83E-16
LDHAP5	0.305	6.55E-16
HEXA	0.367	8.34E-23
AC240504.1	0.383	6.39E-25
CLIC1	0.398	6.02E-27
AP002982.1	0.383	7.66E-25
ACTBP2	0.361	4.52E-22
EIF1P7	0.324	6.94E-18
AL080243.1	0.337	2.80E-19
ITGA1	0.327	3.20E-18
AC078899.1	0.325	5.75E-18
AC091807.1	0.421	3.33E-30
TSPAN4	0.396	1.03E-26
AL390728.1	0.301	1.55E-15
ALG3	0.383	7.30E-25
PTMAP5	0.321	1.49E-17
RPS4XP1	0.313	1.07E-16
LGMNP1	0.381	1.16E-24
NPS	0.325	5.10E-18

LINC02085	0.313	1.04E-16
RPL7P1	0.353	3.67E-21
RPS4XP3	0.308	2.84E-16
IFRD2	0.316	4.33E-17
ZFYVE9P2	0.331	1.33E-18
PKMP5	0.34	1.11E-19
XRCC6P5	0.339	1.69E-19
GPX1P2	0.307	4.28E-16
RCN1P1	0.317	3.50E-17
CNN2P7	0.361	3.83E-22
AL354702.1	0.417	1.20E-29
IFI30	0.366	1.06E-22
ANXA2P3	0.381	1.37E-24
RPS2P55	0.309	2.31E-16
RPL10P1	0.332	9.64E-19
TPT1P4	0.322	1.17E-17
PPIAP31	0.308	3.05E-16
AC136632.1	0.309	2.24E-16
RPS4XP7	0.301	1.47E-15
AL592429.1	0.379	2.36E-24
AL391903.1	0.326	3.87E-18
AC005102.1	0.32	2.05E-17
RPS20P2	0.322	1.00E-17
AL357515.1	0.351	7.39E-21
AC019129.1	0.423	1.40E-30
RPL23P8	0.301	1.53E-15
FTH1P8	0.339	1.65E-19
AL356432.2	0.313	9.01E-17
RPL7P27	0.308	2.79E-16
ACTBP8	0.349	1.00E-20
AL032822.1	0.304	7.59E-16
OSTCP4	0.333	6.88E-19
EBF2	0.324	7.43E-18
AC112721.1	0.748	1.57E-121
AC112721.2	0.719	7.25E-108
POTEJ	0.332	1.03E-18
CYTOR	0.318	2.87E-17
FTH1P10	0.349	1.12E-20
LINC01615	0.33	1.75E-18
TMSB4XP4	0.306	5.10E-16
AC002463.1	0.354	2.66E-21
IGHV3-64	0.313	9.31E-17
AC023157.1	0.317	4.22E-17
AC006145.1	0.419	6.12E-30
RPS20P14	0.362	3.53E-22
LINC01870	0.343	5.99E-20
AC002486.1	0.308	3.49E-16
AC116917.1	0.358	9.72E-22
AL353616.1	0.354	2.86E-21
LINC02561	0.317	3.67E-17
ARSEP1	0.436	1.48E-32

AC104823.1	0.42	3.61E-30
MIR3681HG	0.311	1.53E-16
SMIM25	0.39	7.03E-26
AP001476.1	0.645	2.89E-80
RPL29P19	0.304	7.64E-16
HSPA8P7	0.33	1.42E-18
AC006026.1	0.302	1.34E-15
UBE2D3P1	0.338	2.03E-19
LINC00337	0.438	7.93E-33
PPIAP8	0.331	1.21E-18
RPL8P2	0.329	1.96E-18
RBMS1P1	0.343	5.29E-20
BX119917.1	0.367	7.69E-23
AL358942.1	0.31	2.07E-16
AL121871.1	0.331	1.19E-18
ABHD11-AS1	0.359	6.50E-22
AP001434.1	0.308	3.39E-16
SEC11B	0.337	2.49E-19
RPL26P19	0.315	6.60E-17
ARPC3P1	0.314	7.03E-17
GAPDHP32	0.312	1.37E-16
AC018735.1	0.321	1.57E-17
FTH1P20	0.315	5.55E-17
FTLP3	0.376	4.87E-24
TRBV2	0.319	2.61E-17
AC073409.1	0.315	5.41E-17
HNRNPA3P1	0.309	2.39E-16
AC011893.1	0.541	2.57E-52
AL117382.1	0.461	1.17E-36
RPS4XP2	0.329	2.22E-18
AF130417.1	0.472	1.47E-38
FTH1P16	0.324	7.07E-18
AP001471.1	0.429	1.80E-31
HNRNPA1P21	0.405	6.40E-28
AP001476.3	0.39	7.35E-26
LYPLA2P1	0.311	1.67E-16
AL158201.1	0.302	1.36E-15
FTLP17	0.353	4.19E-21
AL109741.2	0.323	9.44E-18
TMSB10P1	0.398	6.58E-27
LYPLAL1-AS	0.652	1.60E-82
AL513323.1	0.459	2.52E-36
LINC02577	0.47	2.65E-38
AL359918.1	0.324	6.29E-18
AC006483.1	0.433	4.48E-32
HECW2-AS1	0.528	1.81E-49
EIF4A1P10	0.347	1.69E-20
ACTBP1	0.339	1.41E-19
SERPINH1P1	0.546	1.57E-53
ACTG1P9	0.31	1.88E-16
LINC01204	0.498	2.60E-43

AL109810.1	0.311	1.65E-16
MYL8P	0.318	2.66E-17
RPL4P4	0.302	1.17E-15
MYOSLID	0.336	3.05E-19
RPL7P10	0.319	2.31E-17
AC018866.1	0.582	3.26E-62
ELOCP3	0.362	3.06E-22
HSPD1P6	0.362	2.75E-22
AC139143.1	0.323	8.95E-18
AC016738.2	0.36	5.12E-22
AC018463.1	0.323	8.29E-18
AC006970.1	0.37	2.92E-23
DDX43P3	0.433	4.59E-32
AL450405.1	0.351	7.00E-21
FTH1P5	0.308	3.13E-16
HSPB1P2	0.387	2.08E-25
BX088651.3	0.332	8.70E-19
NFE4	0.359	7.36E-22
AC078817.1	0.316	5.33E-17
ACTG1P23	0.303	9.99E-16
LINC01747	0.507	4.18E-45
AP000695.1	0.366	9.33E-23
ACTG1P14	0.304	7.56E-16
GPAA1P2	0.301	1.68E-15
PABPC1P3	0.359	7.80E-22
AL450003.1	0.308	3.31E-16
AL161636.2	0.309	2.35E-16
AC068580.2	0.336	3.81E-19
LINC01614	0.316	4.72E-17
AC013404.1	0.309	2.59E-16
SPATA20P1	0.338	2.03E-19
TUBB4BP6	0.376	5.57E-24
ARF4P4	0.307	3.84E-16
SBK3	0.466	1.31E-37
MANCR	0.4	3.56E-27
CLIC1P1	0.387	2.26E-25
AC105450.1	0.501	6.43E-44
LINC02015	0.346	2.43E-20
EIF1P3	0.337	2.59E-19
PABPC1P1	0.398	6.35E-27
EMBP1	0.328	2.76E-18
AC012354.2	0.322	1.02E-17
AC073316.2	0.427	4.42E-31
AC024082.2	0.384	5.50E-25
ANXA2P2	0.463	5.96E-37
CAP1P2	0.321	1.53E-17
LEF1-AS1	0.304	8.39E-16
AP001057.1	0.325	5.11E-18
P2RY10BP	0.418	7.62E-30
FTH1P7	0.327	3.19E-18
SLC25A6P2	0.348	1.66E-20

IGHV3-43	0.324	7.33E-18
FTH1P1	0.322	1.13E-17
AC009299.2	0.326	3.99E-18
AC093627.1	0.589	6.14E-64
Z74021.1	0.332	9.45E-19
FTLP2	0.377	4.15E-24
AL583856.1	0.339	1.57E-19
EEF1B2P3	0.304	8.37E-16
RPL3P12	0.314	8.12E-17
POLR3KP1	0.308	3.31E-16
ACTG1P21	0.313	9.79E-17
TRIM51JP	0.477	1.83E-39
AL645568.3	0.305	6.17E-16
ITGA6-AS1	0.303	9.60E-16
SDCBPP3	0.326	4.07E-18
TRBV29-1	0.325	5.93E-18
CYP1B1-AS1	0.489	1.11E-41
AC011899.2	0.322	1.14E-17
PFN1P1	0.372	1.53E-23
ERP29P1	0.355	2.38E-21
RPS2P48	0.312	1.11E-16
AL445524.1	0.327	2.93E-18
AC008163.1	0.436	1.57E-32
LINC00460	0.55	2.55E-54
TWIST2	0.678	9.05E-92
PSMA6P3	0.588	8.14E-64
AP000695.2	0.339	1.63E-19
LINC02599	0.515	1.00E-46
LINC01503	0.323	9.16E-18
FTLP15	0.304	8.16E-16
RPS20P10	0.332	9.23E-19
GDI2P2	0.311	1.56E-16
TXNP6	0.302	1.23E-15
RPL10P12	0.303	1.08E-15
OSTCP5	0.322	1.23E-17
RPS4XP11	0.334	6.08E-19
AC026462.1	0.426	6.02E-31
AL162151.2	0.313	8.63E-17
LINC01934	0.339	1.58E-19
TPT1P9	0.309	2.56E-16
SVIL2P	0.366	1.03E-22
XRCC6P2	0.335	4.04E-19
FTH1P2	0.336	3.63E-19
EIF4A1P2	0.341	9.23E-20
TMSB10P2	0.379	2.51E-24
RPEL1	0.315	6.26E-17
AC113174.1	0.44	3.24E-33
EIF4A1P7	0.351	6.81E-21
RNF148	0.306	4.54E-16
RPL10P5	0.318	3.24E-17
MXRA5Y	0.696	1.27E-98

LINC01936	0.405	5.84E-28
PRRX2-AS1	0.313	9.75E-17
HSPB1P1	0.388	1.33E-25
AC026355.2	0.696	1.87E-98
PKMP1	0.361	4.35E-22
AC003986.3	0.411	8.61E-29
AC116049.1	0.339	1.63E-19
RAD23BP1	0.316	4.65E-17
S100A11P2	0.463	4.71E-37
EIF4A1P6	0.37	2.75E-23
RPL19P16	0.327	3.40E-18
AC007750.1	0.368	5.89E-23
RPS20P24	0.308	3.01E-16
TMSB4XP1	0.311	1.53E-16
NDUFA5P8	0.385	3.62E-25
AC147651.4	0.393	2.68E-26
IMPDH1P4	0.351	5.74E-21
TRBV30	0.309	2.67E-16
FTH1P11	0.325	5.59E-18
CDC42P6	0.413	4.63E-29
AC034102.1	0.361	4.49E-22
AC007384.1	0.483	1.76E-40
S100A11P1	0.427	4.13E-31
TRBV3-1	0.356	1.76E-21
AC097713.2	0.362	3.39E-22
AC002066.1	0.369	3.74E-23
PA2G4P1	0.334	5.36E-19
AC073130.1	0.68	2.03E-92
AC245884.4	0.349	1.01E-20
ACTG1P19	0.336	3.20E-19
AL391832.2	0.376	6.15E-24
AP000844.2	0.521	6.27E-48
AC008753.1	0.327	3.28E-18
TXNDC5	0.565	5.97E-58
AC108724.1	0.308	3.00E-16
RPL37P2	0.338	1.82E-19
IGKV2D-30	0.348	1.57E-20
APOBEC3G	0.335	4.29E-19
AC093627.2	0.487	2.14E-41
AL133163.1	0.332	9.17E-19
AC093627.3	0.438	7.04E-33
AC017002.3	0.322	1.09E-17
AC010343.1	0.305	5.78E-16
LINC00973	0.691	1.92E-96
RPL3P9	0.304	8.19E-16
RPL30P12	0.302	1.29E-15
C1QTNF9	0.461	9.65E-37
AC093627.4	0.489	1.26E-41
IGKV1-16	0.312	1.23E-16
AC021074.1	0.351	6.22E-21
RPL22P2	0.309	2.29E-16

RPL17P44	0.318	2.74E-17
AC245047.8	0.32	1.73E-17
RPL7P19	0.32	1.64E-17
HSPA8P9	0.315	6.53E-17
AL355032.1	0.326	4.28E-18
ARPC1A	0.386	2.37E-25
LINC02657	0.331	1.12E-18
AC113398.1	0.337	2.42E-19
FTH1P23	0.327	3.33E-18
FTH1P4	0.327	2.95E-18
AC079760.2	0.363	2.36E-22
AC007686.1	0.358	8.50E-22
IGKV2-30	0.322	1.18E-17
IGKV2D-29	0.301	1.38E-15
EFNA4	0.325	4.76E-18
RPL3P3	0.312	1.19E-16
TICAM2	0.32	1.99E-17
IL10RB	0.45	8.11E-35
RPS4XP14	0.303	9.33E-16
RPLP0P2	0.401	2.36E-27
TMEM35B	0.384	4.47E-25
APOBEC3D	0.362	3.11E-22
RPL12P6	0.328	2.59E-18
AC073359.2	0.332	1.06E-18
DDOST	0.339	1.50E-19
RPS4XP17	0.304	7.15E-16
AC133134.1	0.353	3.33E-21
AC147067.1	0.439	5.66E-33
LILRA6	0.349	1.02E-20
APOBEC3C	0.409	1.54E-28
RPL12P33	0.326	3.85E-18
CASC8	0.471	2.43E-38
AC100861.1	0.458	4.28E-36
LINC00920	0.404	1.01E-27
TWF2	0.349	1.07E-20
ADH1C	0.469	4.21E-38
APELA	0.506	5.06E-45
LINC02434	0.583	2.40E-62
AC187653.1	0.301	1.44E-15
AC098859.1	0.301	1.51E-15
AC131254.1	0.531	3.45E-50
AC093895.1	0.304	7.92E-16
ADAM20P3	0.424	1.26E-30
LINC01091	0.394	2.12E-26
SERBP1P5	0.305	6.41E-16
AC099509.1	0.395	1.65E-26
AC145676.1	0.385	3.91E-25
AC239584.1	0.377	4.04E-24
AC093791.1	0.311	1.63E-16
AC069360.1	0.379	1.94E-24
AC116563.1	0.318	3.09E-17

AC079140.2	0.305	6.80E-16
GMPSP1	0.314	6.98E-17
LINC01303	0.323	7.88E-18
AC098591.2	0.411	1.06E-28
AC097451.1	0.383	6.41E-25
AC146944.2	0.324	6.42E-18
AC010343.3	0.497	3.10E-43
AC080079.1	0.348	1.65E-20
AC105250.1	0.398	6.16E-27
AC025470.2	0.419	6.08E-30
AC020551.1	0.397	8.14E-27
AC091173.1	0.646	1.12E-80
LDHAP1	0.32	2.04E-17
MSNP1	0.34	1.15E-19
LINC02278	0.338	2.06E-19
RF00397	0.367	8.10E-23
AC004707.1	0.443	1.12E-33
LINC01605	0.425	7.06E-31
AC109479.1	0.397	7.55E-27
NRBF2P4	0.31	1.82E-16
TMEM200B	0.478	1.13E-39
AC016868.1	0.422	1.87E-30
AC022915.1	0.621	5.98E-73
SEPT10P1	0.317	3.46E-17
AC107959.3	0.328	2.79E-18
TAGLN2P1	0.367	7.72E-23
AC026904.2	0.327	3.67E-18
AC026904.3	0.537	1.45E-51
AC091182.2	0.354	2.99E-21
IGLV1-41	0.531	3.29E-50
TNFRSF10A-	0.346	2.36E-20
HMGN1P38	0.318	2.79E-17
CLDN23	0.553	5.38E-55
PKMP4	0.362	2.85E-22
LINC01947	0.308	3.37E-16
AC087672.2	0.416	1.42E-29
AF201337.1	0.451	6.21E-35
MYL12AP1	0.312	1.11E-16
LINC02584	0.67	1.16E-88
SIGLEC12	0.621	4.80E-73
AP003555.2	0.768	9.87E-132
AC080023.2	0.342	7.47E-20
OR2AL1P	0.459	2.18E-36
AC023232.1	0.373	1.43E-23
SLC22A18AS	0.313	9.88E-17
AC113404.3	0.314	8.44E-17
AP003386.1	0.513	2.29E-46
AC108136.1	0.405	7.35E-28
TRAV1-1	0.341	8.49E-20
LINC00944	0.33	1.49E-18
LINC02454	0.405	6.92E-28

LINC02376	0.353	4.09E-21
AC092470.1	0.393	3.37E-26
HSPA8P5	0.324	7.66E-18
RPL31P57	0.357	1.19E-21
AP005019.1	0.402	1.50E-27
AP002784.2	0.314	7.92E-17
AC090023.2	0.315	6.26E-17
AP001453.2	0.337	2.51E-19
AC026369.3	0.325	5.12E-18
RARSP1	0.341	9.97E-20
AP003174.3	0.339	1.55E-19
AC009729.1	0.476	2.54E-39
LINC02354	0.38	1.61E-24
AC073611.2	0.435	2.23E-32
LBX2-AS1	0.444	8.74E-34
EIF4A1P4	0.363	2.59E-22
HMGA1P3	0.327	3.01E-18
SLC25A3P2	0.304	8.27E-16
RBM8B	0.323	8.15E-18
EIF3LP1	0.301	1.58E-15
LINC00239	0.456	7.55E-36
LINC00520	0.466	1.68E-37
AL355102.4	0.396	1.02E-26
LINC02289	0.307	3.87E-16
AC104390.1	0.336	3.78E-19
AL049775.2	0.685	2.40E-94
AL135978.1	0.315	5.39E-17
AC104002.3	0.301	1.57E-15
ITGB3	0.354	2.82E-21
LINC02345	0.415	2.51E-29
IGHV4OR15-	0.306	4.96E-16
AC026956.1	0.333	7.33E-19
HSP90B2P	0.379	2.33E-24
AC009093.1	0.419	6.65E-30
LINC02126	0.317	4.21E-17
AL121578.3	0.309	2.75E-16
MRC1	0.378	3.22E-24
MOCS1P1	0.318	2.84E-17
AC009093.2	0.411	9.70E-29
AC023043.1	0.494	1.03E-42
FOXC2-AS1	0.344	4.63E-20
AC004847.1	0.412	5.65E-29
AC113418.1	0.319	2.41E-17
LMO7-AS1	0.422	1.92E-30
LINC01989	0.486	4.55E-41
AC134312.5	0.49	5.87E-42
PECAM1	0.336	3.59E-19
AC092368.3	0.318	2.86E-17
FSCN1P1	0.317	3.56E-17
AP003119.3	0.342	7.08E-20
LOXL1-AS1	0.349	1.13E-20

AC087392.1	0.309	2.77E-16
AC129507.4	0.316	4.51E-17
AC022035.1	0.638	3.50E-78
AC005291.1	0.514	1.16E-46
AC116003.1	0.486	4.20E-41
LINC01910	0.309	2.69E-16
AC091588.3	0.356	1.87E-21
GAPLINC	0.377	4.54E-24
ACTBP9	0.343	5.29E-20
AC005262.1	0.324	7.56E-18
FTLP5	0.355	2.00E-21
LINC01929	0.426	4.82E-31
AC090409.1	0.347	2.00E-20
AC010649.1	0.427	3.28E-31
AC079210.1	0.426	5.88E-31
AC010327.3	0.307	3.96E-16
LINC01482	0.413	4.96E-29
AC098847.1	0.322	1.00E-17
AC093567.1	0.364	1.65E-22
AC243960.2	0.393	3.43E-26
AC010328.2	0.371	2.35E-23
CKS1BP3	0.312	1.25E-16
LYPLA2P2	0.352	4.73E-21
AC010422.3	0.325	5.54E-18
AC022149.1	0.535	5.58E-51
AC008763.3	0.312	1.19E-16
AL353593.1	0.373	1.13E-23
IGHV1OR15-	0.311	1.58E-16
IGHV3-30	0.321	1.36E-17
NBPF14	0.35	8.45E-21
AL606490.9	0.355	1.98E-21
PDLIM1P1	0.361	4.61E-22
IGHV3OR16-	0.334	5.57E-19
AC009948.3	0.44	3.72E-33
CTHRC1P1	0.481	2.88E-40
IGHV3OR16-	0.359	6.38E-22
AC012498.2	0.313	1.08E-16
IGHV3OR16-	0.411	7.79E-29
LINC01050	0.535	4.38E-51
AL683887.1	0.397	9.99E-27
LINC02104	0.306	5.24E-16
CCL5	0.385	3.65E-25
ARF4P1	0.367	6.88E-23
ATP1B3P1	0.324	6.09E-18
AC017076.1	0.324	7.47E-18
AL008726.1	0.354	2.98E-21
AC147067.2	0.325	5.30E-18
AC011899.3	0.404	8.70E-28
AC006033.2	0.32	1.82E-17
AL365203.2	0.444	6.73E-34
AC005046.1	0.384	4.33E-25

AC108673.2	0.406	5.14E-28
AC005291.2	0.512	3.66E-46
AP000904.1	0.615	4.73E-71
MIR6511A4	0.746	2.66E-120
AC100757.1	0.342	7.92E-20
BX322562.1	0.416	1.72E-29
RIMBP3B	0.375	6.28E-24
CCL23	0.349	1.02E-20
F8A2	0.323	9.42E-18
AC023043.4	0.329	2.12E-18
AC009804.1	0.338	2.29E-19
AC067904.3	0.333	7.55E-19
LENG9	0.391	4.96E-26
LINC00547	0.437	1.00E-32
AC004816.2	0.417	1.23E-29
RIMBP3	0.303	8.82E-16
AC244153.1	0.307	4.30E-16
AC243829.4	0.304	7.73E-16
F8A3	0.301	1.54E-15
ATP5F1AP10	0.36	6.00E-22
AL357033.4	0.325	5.44E-18
RF02133	0.369	4.35E-23
AC087633.2	0.426	5.06E-31
AC239859.6	0.301	1.54E-15
TRAC	0.436	1.60E-32
AC121338.2	0.309	2.28E-16
AC079298.1	0.328	2.59E-18
AC073130.3	0.526	4.93E-49
AL118508.2	0.331	1.21E-18
AC018629.1	0.356	1.57E-21
AC041040.1	0.361	4.12E-22
AC010894.5	0.343	5.46E-20
AP003485.2	0.391	4.93E-26
AC005831.1	0.305	5.65E-16
LINC01101	0.342	7.04E-20
LINC01943	0.412	5.94E-29
LINC01173	0.478	1.00E-39
