# Supplementary material for: CD13 expression affects glioma patient survival and influences key functions of human glioblastoma cell lines in vitro
Source: BMC Cancer. 2024 Mar 22;24:369. doi: 10.1186/s12885-024-12113-z (PMC10960415; doi:10.1186/s12885-024-12113-z)
Supplement: Supplementary file 5 — Supplementary Material 5 [file 12885_2024_12113_MOESM5_ESM.pdf]

Supplementary Table 5. *CD13* correlated with collagens and MMPs

Gene	r	P
Collagens		
COL1A2	0.805	3.83E-154
COL3A1	0.794	3.05E-147
COL1A1	0.783	2.47E-140
COL6A3	0.781	7.53E-139
COL5A1	0.774	5.26E-135
COL6A2	0.769	4.33E-132
COL4A1	0.692	5.63E-97
COL4A2	0.69	2.71E-96
COL5A2	0.679	4.58E-92
COL18A1	0.674	4.61E-90
COL15A1	0.649	1.80E-81
COL6A1	0.639	2.03E-78
COL8A1	0.558	3.29E-56
COL13A1	0.522	3.27E-48
COL12A1	0.492	3.31E-42
COL14A1	0.409	2.03E-28
COL4A2-AS1	0.386	2.40E-25
COL10A1	0.364	1.56E-22
COL5A3	0.335	4.95E-19
COL8A2	0.321	1.44E-17
COL22A1	0.317	4.14E-17
COL11A2	-0.343	4.89E-20
MMPs		
MMP9	0.708	3.67E-103
MMP11	0.695	5.01E-98
MMP19	0.685	2.43E-94
MMP1	0.626	1.51E-74
MMP14	0.569	5.76E-59
MMP7	0.536	3.15E-51
MMP2	0.51	8.83E-46
MMP8	0.494	1.26E-42
MMP13	0.48	5.53E-40
MMP25	0.478	9.88E-40
MMP10	0.362	3.05E-22
MMP12	0.353	3.85E-21
MMP3	0.337	2.78E-19

COL, Collagen; MMPs, Matrix metalloproteinases; r, correlation coefficient; P, P-value.
