# Supplementary material for: CD13 expression affects glioma patient survival and influences key functions of human glioblastoma cell lines in vitro
Source: BMC Cancer. 2024 Mar 22;24:369. doi: 10.1186/s12885-024-12113-z (PMC10960415; doi:10.1186/s12885-024-12113-z)
Supplement: Supplementary file 6 — Supplementary Material 6 [file 12885_2024_12113_MOESM6_ESM.pdf]

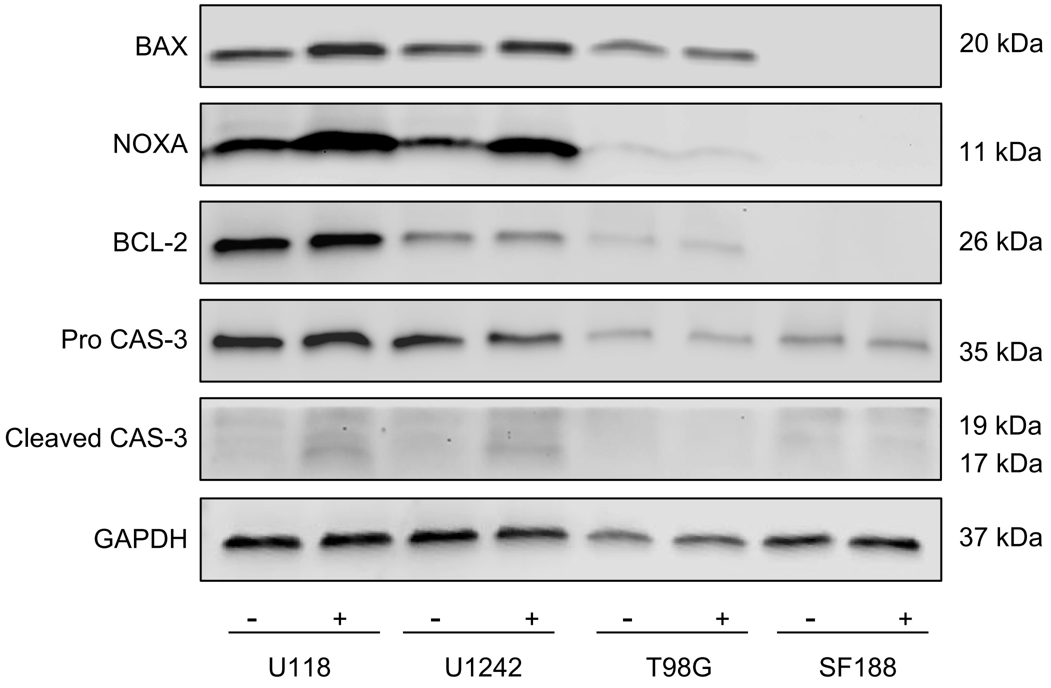

Supplementary Figure 1. Representative western blotting. Example of western blots detecting BAX, BCL-2, NOXA, Pro and Cleaved CAS 3 after 48h of bestatin treatment. GAPDH was used as loading control (-, without drug; +, 500 µg/mL bestatin). Full-length blots are presented in Additional file 7.

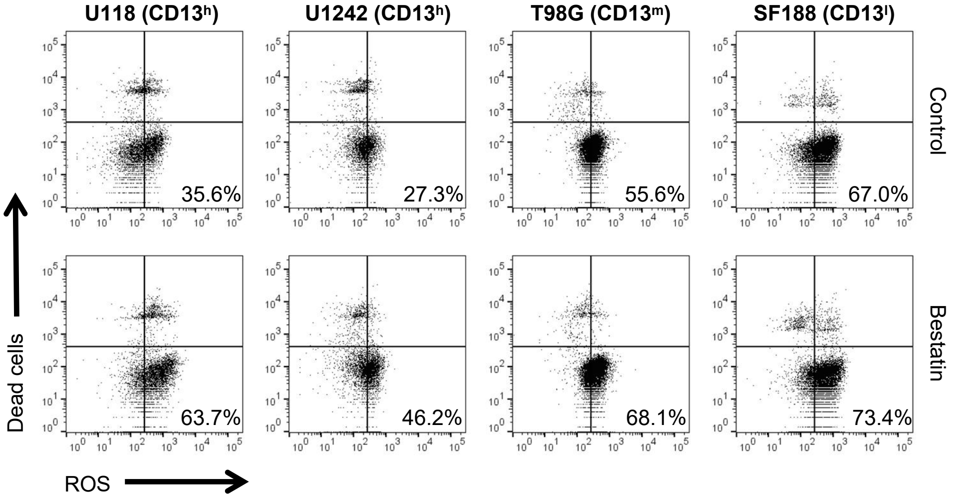

Supplementary Figure 2. Representative dot plot of ROS detection. Following bestatin treatment (500 μg/mL), cells were trypsinized, and cells were incubated with CellROX® Detection Reagent. Representative images of dot plots are shown. Percentages indicate ROS production of living cells. $CD13^h$, high CD13 expression; $CD13^m$, medium CD13 expression; $CD13^l$, low CD13 expression.
