# Supplementary material for: CD13 expression affects glioma patient survival and influences key functions of human glioblastoma cell lines in vitro
Source: BMC Cancer. 2024 Mar 22;24:369. doi: 10.1186/s12885-024-12113-z (PMC10960415; doi:10.1186/s12885-024-12113-z)

CD13 (160 kDa and 130 kDa)—Figure 4A

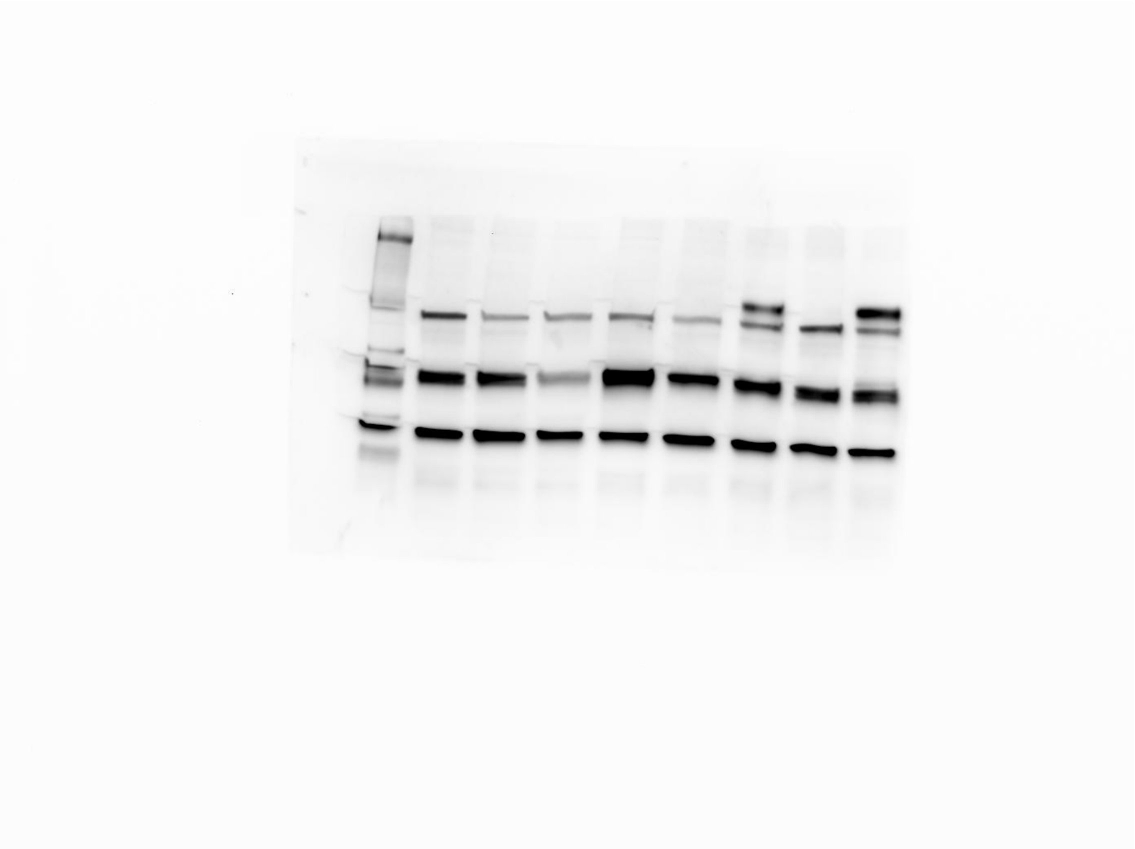

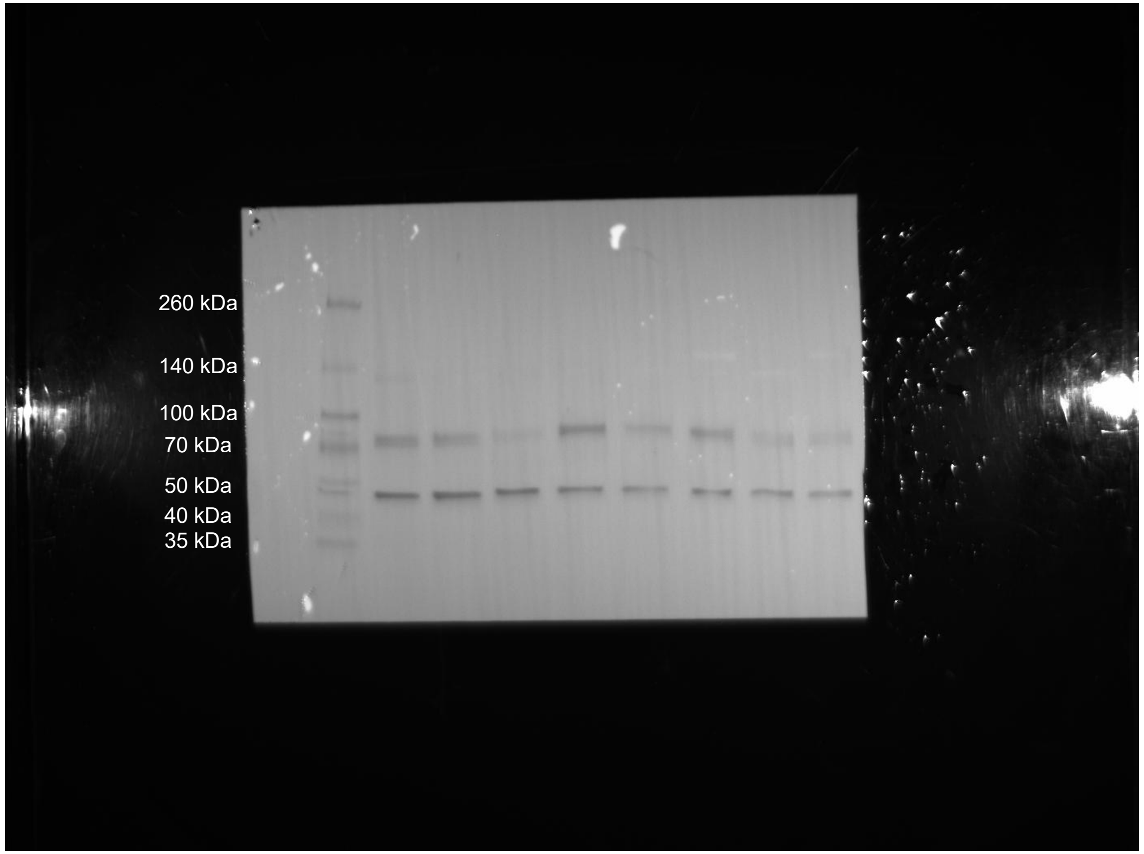

GAPDH (37 kDa)—Figure 4A

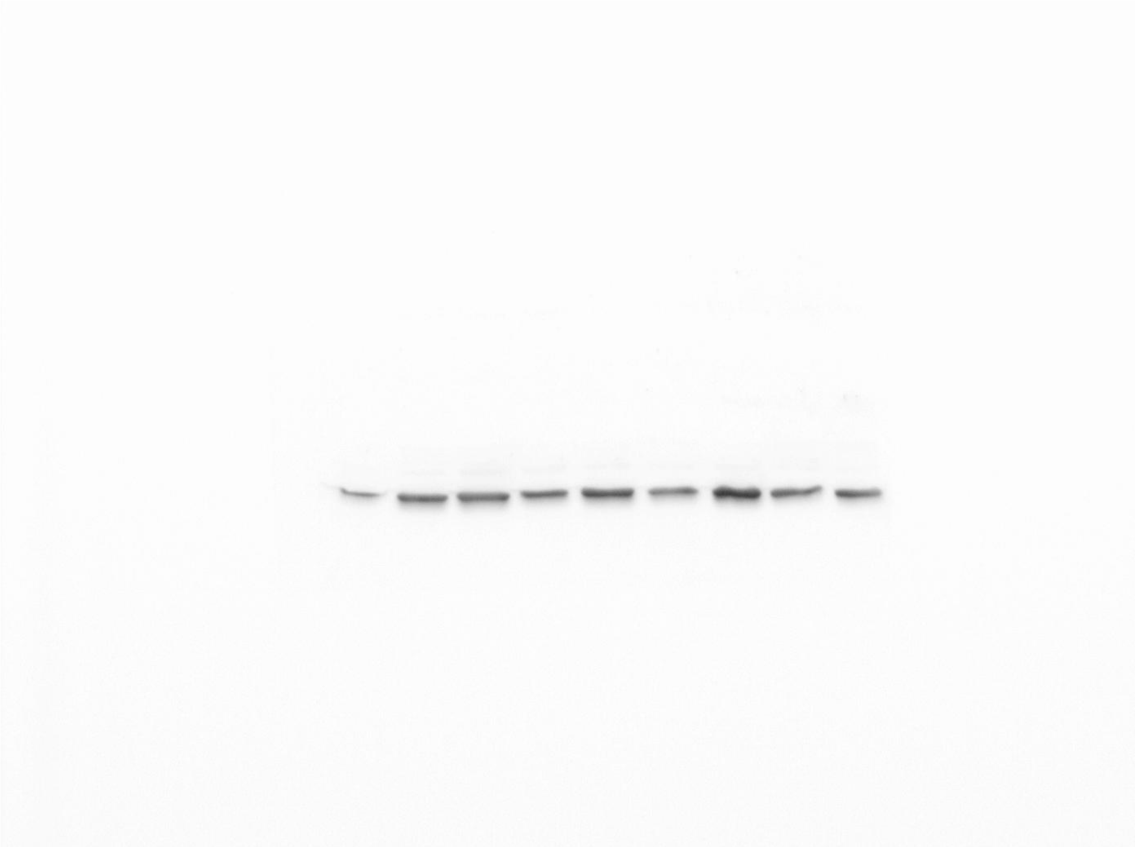

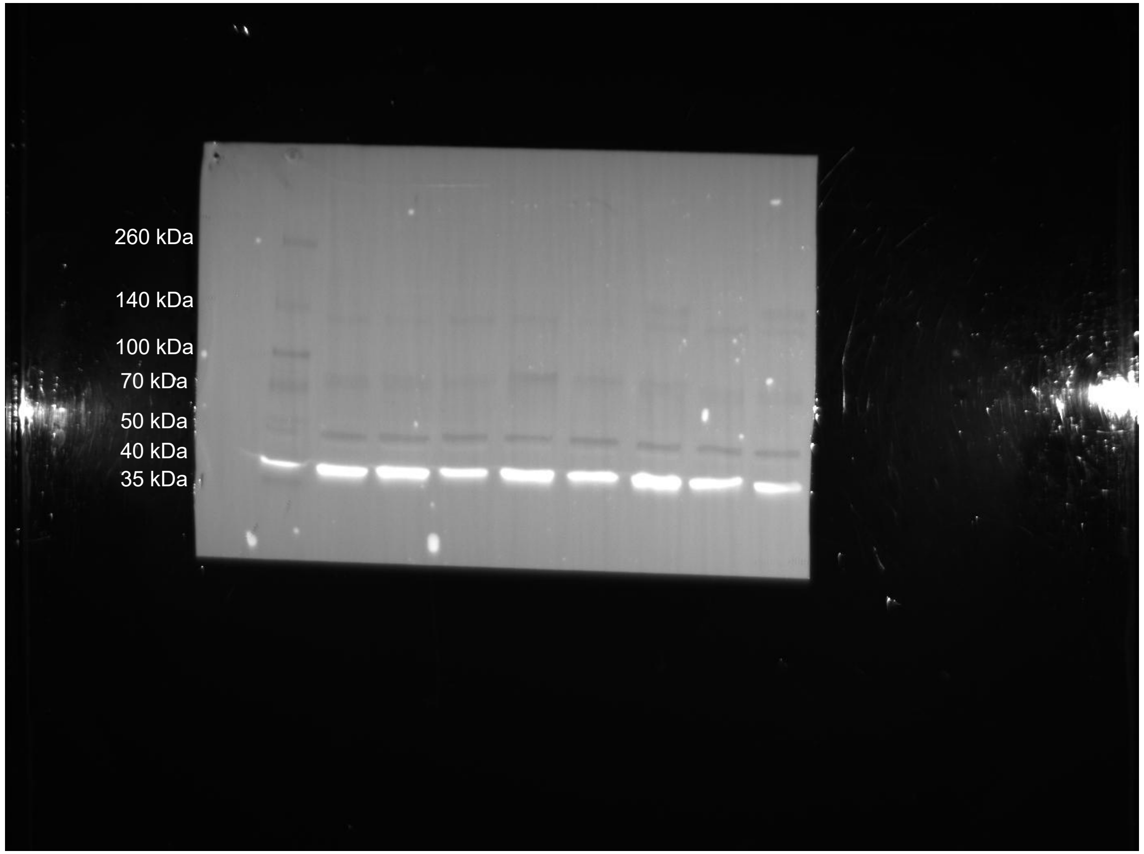

BAX (20 kDa)—Supplementary Figure S1

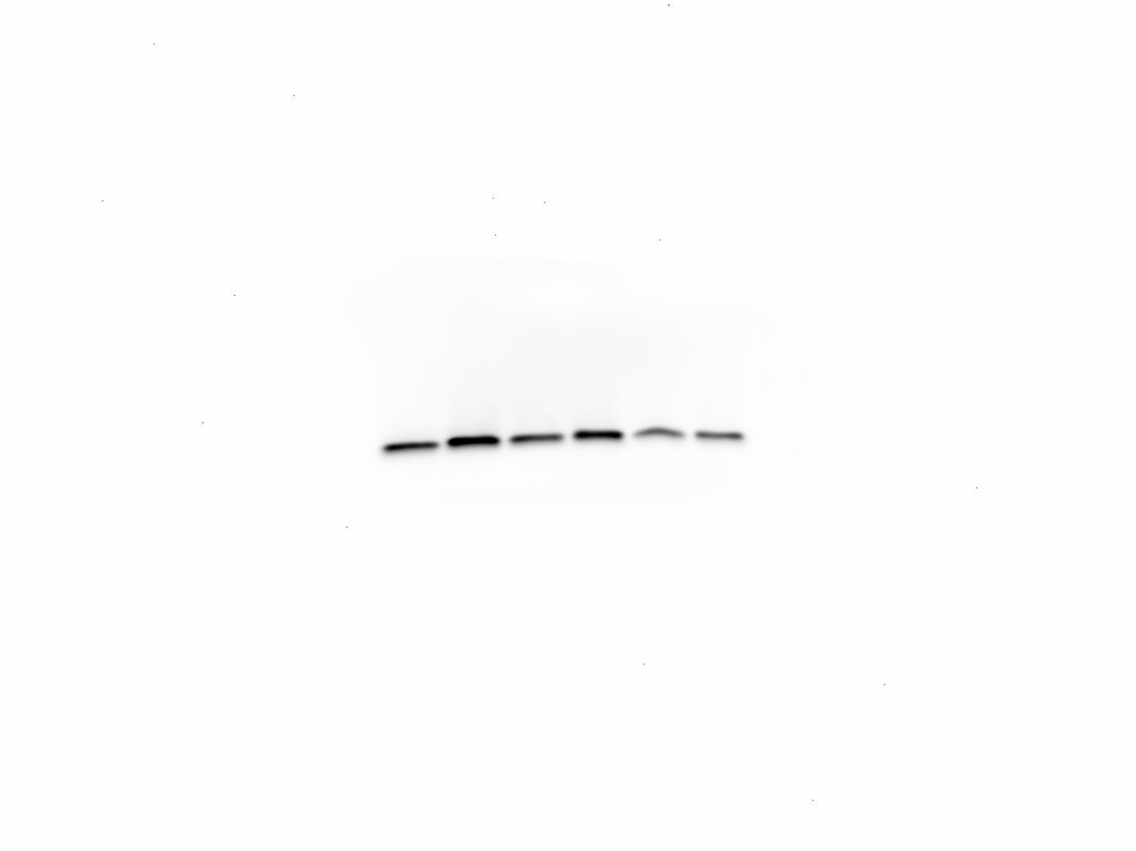

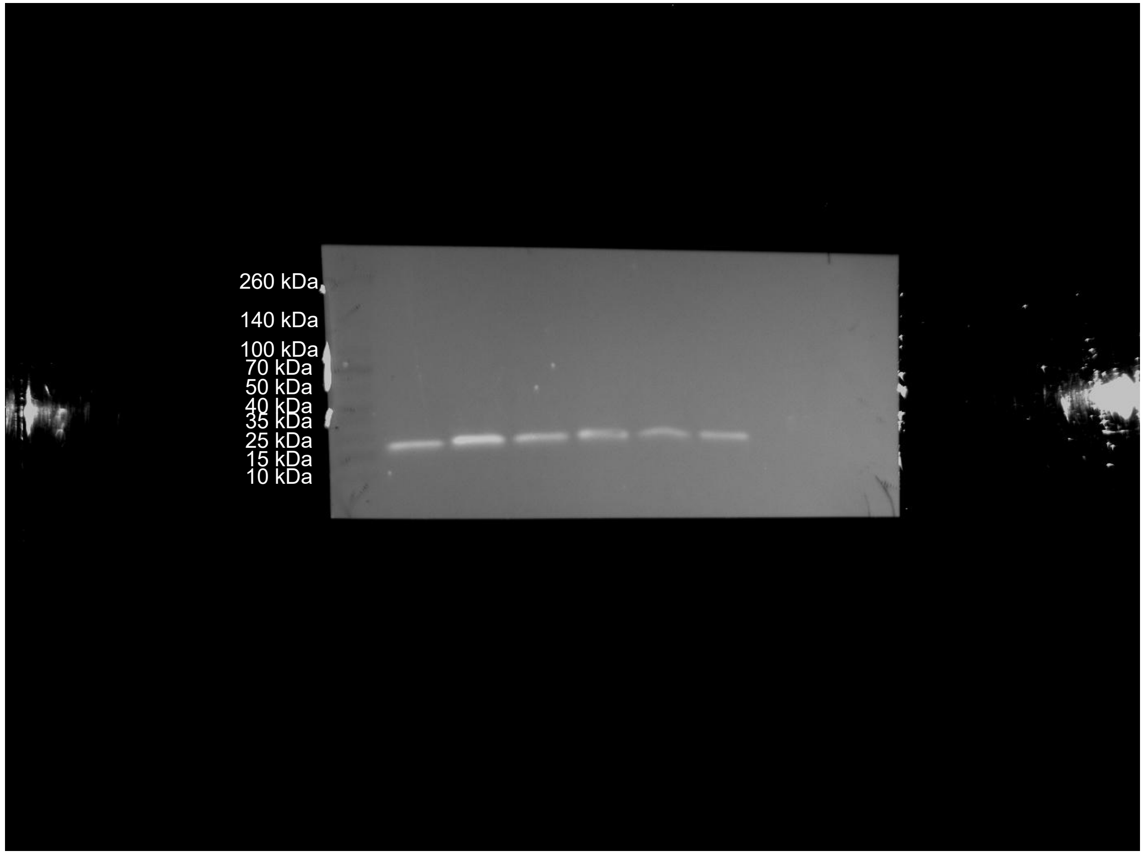

NOXA (11 kDa)—Supplementary Figure S1

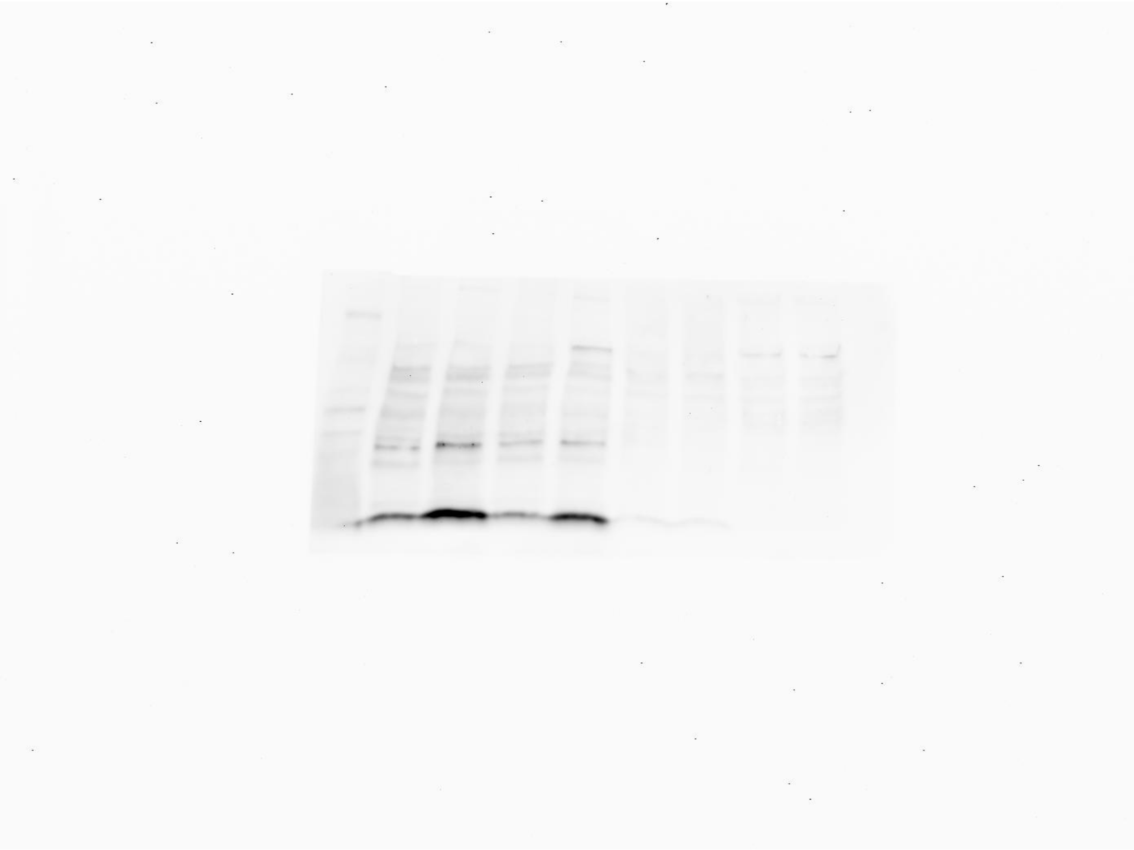

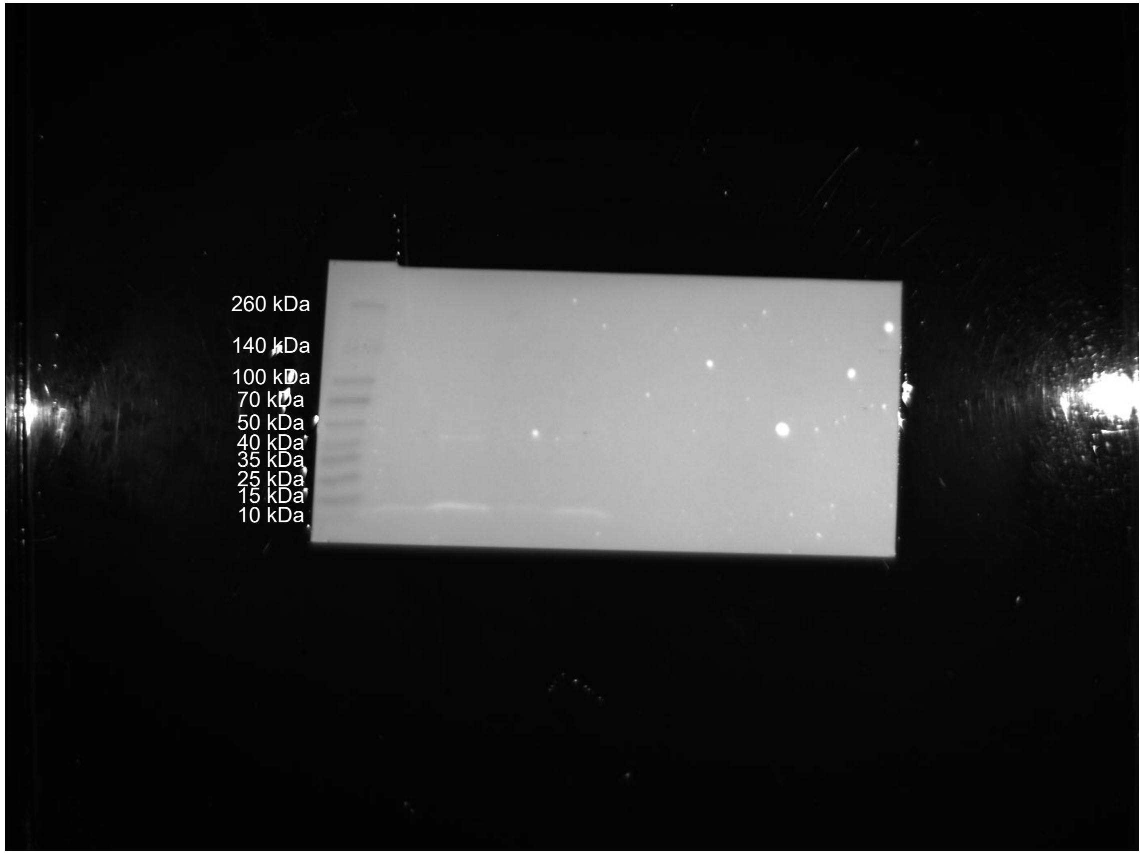

BCL 2 (26 kDa)—Supplementary Figure S1

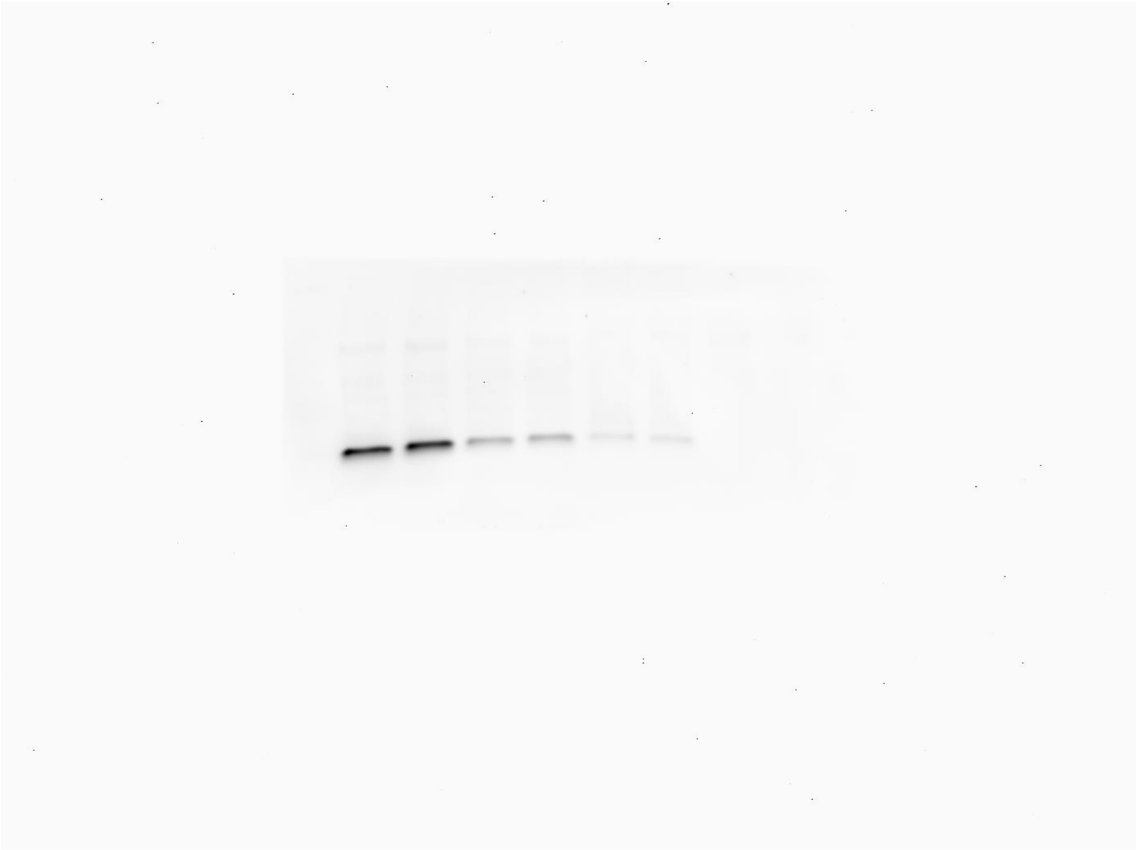

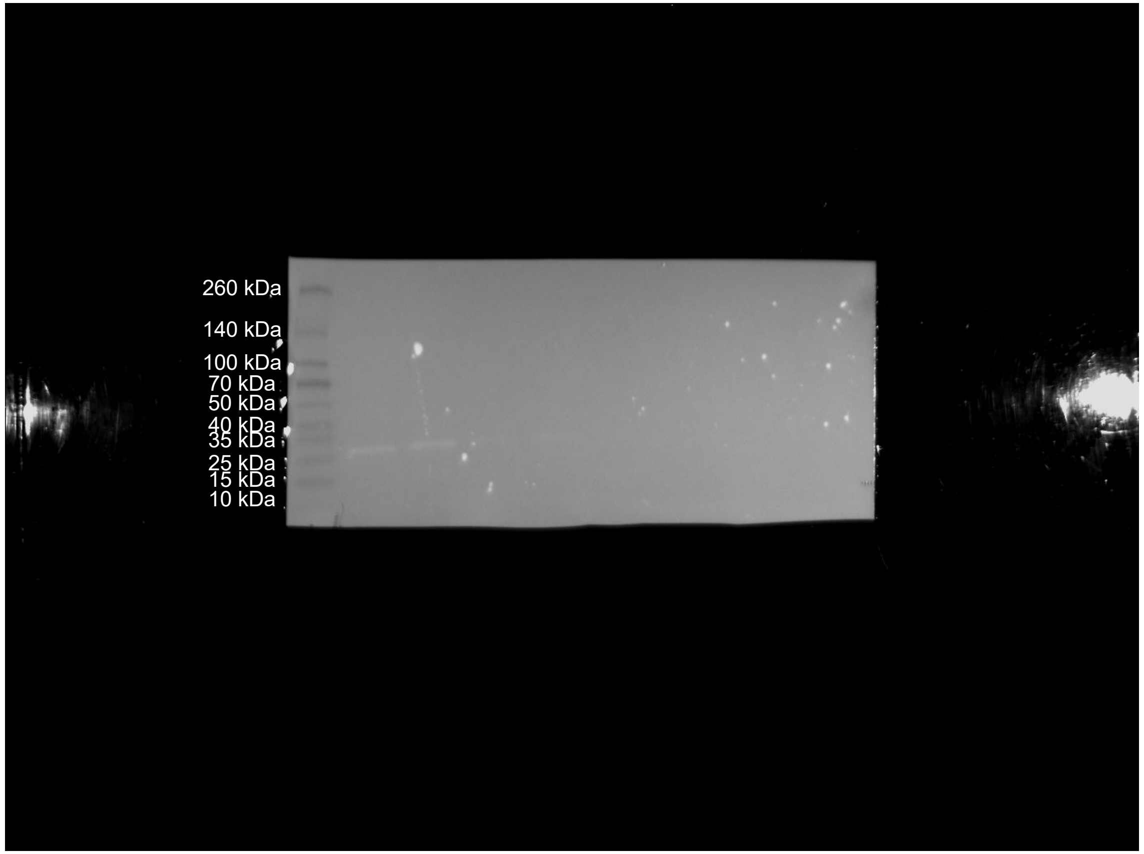

Pro CAS-3 (35 kDa)—Supplementary Figure S1

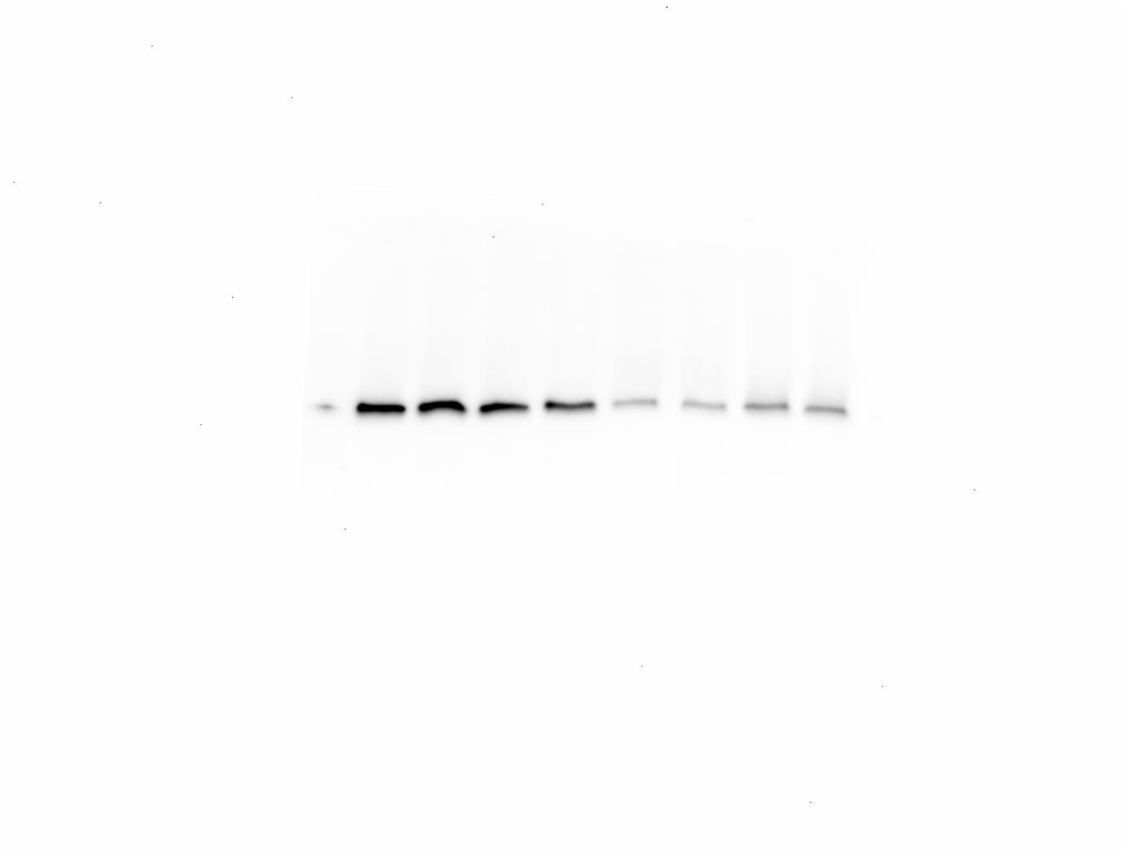

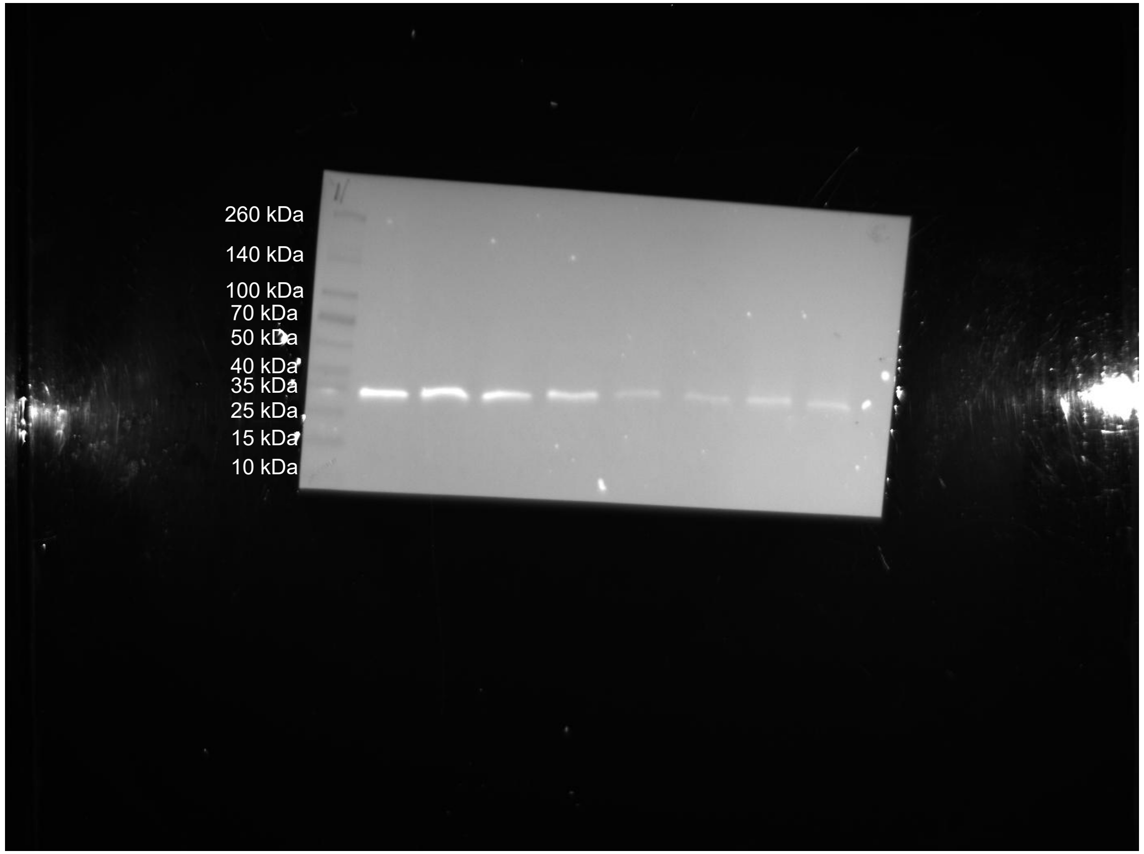

Cleaved CAS-3 (19 kDa and 17 kDa)—Supplementary Figure S1

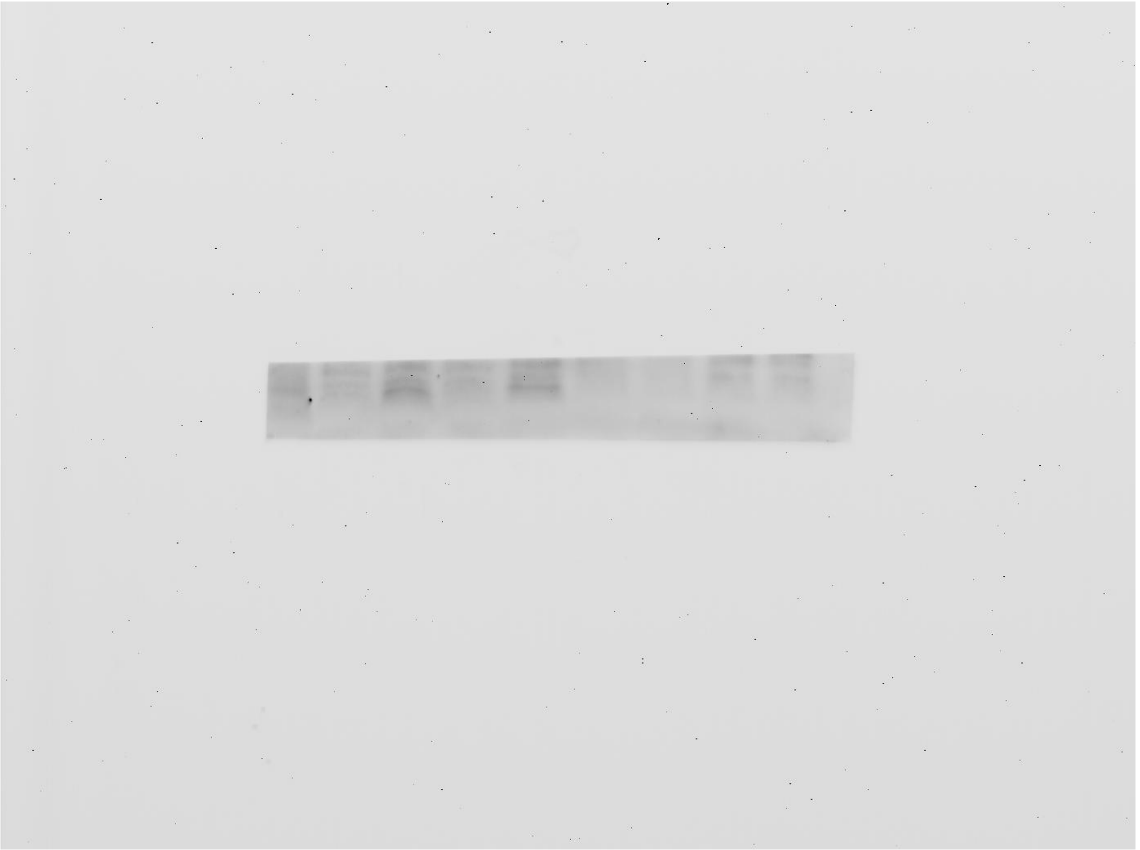

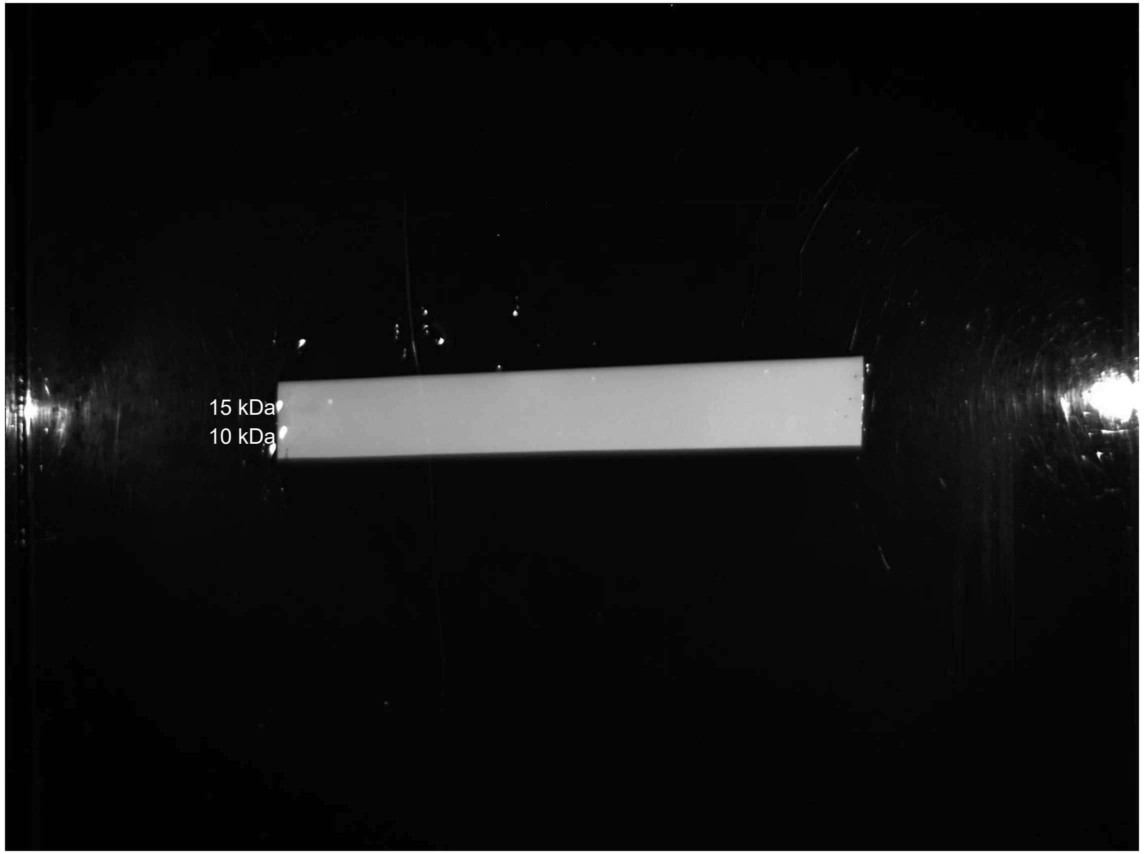

GAPDH (37 kDa)—Supplementary Figure S1

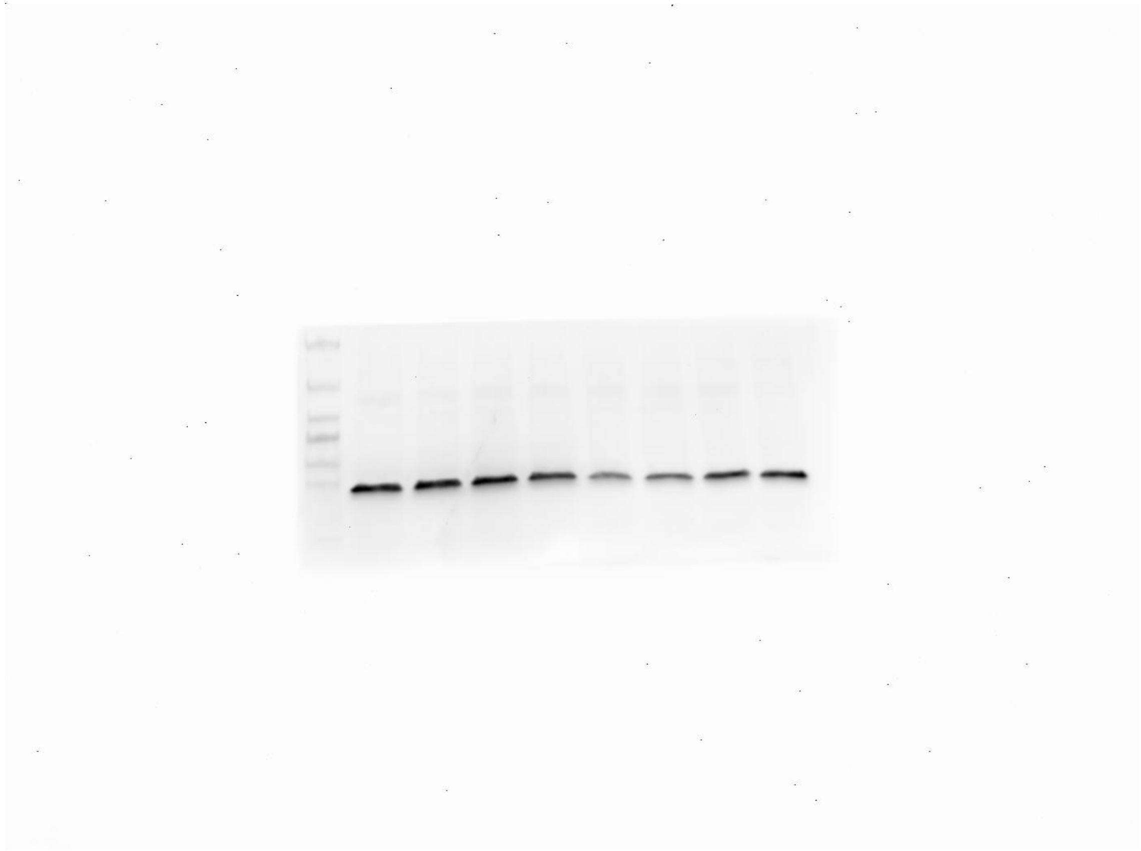

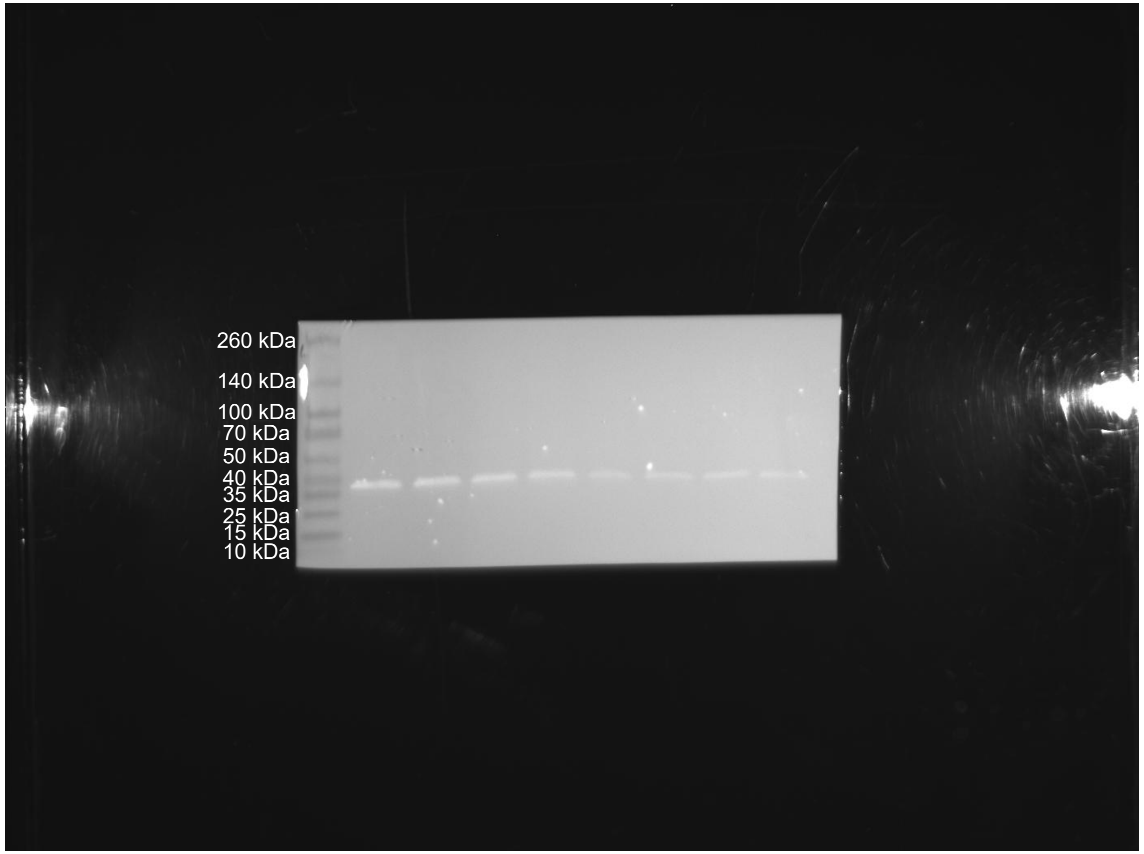

Supplement: Supplementary file 7 — Supplementary Material 7 [file 12885_2024_12113_MOESM7_ESM.pdf]
